# Invited Review Article: Measurements of the Newtonian constant of gravitation, *G*

**DOI:** 10.1063/1.4994619

**Published:** 2017-11

**Authors:** C. Rothleitner, S. Schlamminger

**Affiliations:** 1 Physikalisch-Technische Bundesanstalt (PTB), Bundesallee 100, 38116 Braunschweig, Germany; 2 National Institute of Standards and Technology (NIST), 100 Bureau Drive Stop 8171, Gaithersburg, Maryland 20899, USA; 3 Ostbayerische Technische Hochschule (OTH) Regensburg, Seybothstr. 2, 93053 Regensburg, Germany

## Abstract

By many accounts, the Newtonian constant of gravitation *G* is the fundamental constant that is most difficult to measure accurately. Over the past three decades, more than a dozen precision measurements of this constant have been performed. However, the scatter of the data points is much larger than the uncertainties assigned to each individual measurement, yielding a Birge ratio of about five. Today, *G* is known with a relative standard uncertainty of 4.7 × 10^−5^, which is several orders of magnitudes greater than the relative uncertainties of other fundamental constants. In this article, various methods to measure *G* are discussed. A large array of different instruments ranging from the simple torsion balance to the sophisticated atom interferometer can be used to determine *G*. Some instruments, such as the torsion balance can be used in several different ways. In this article, the advantages and disadvantages of different instruments as well as different methods are discussed. A narrative arc from the historical beginnings of the different methods to their modern implementation is given. Finally, the article ends with a brief overview of the current state of the art and an outlook.

## I. INTRODUCTION

Gravitation is the most tangible interaction of the four fundamental interactions of nature, having been investigated long before the discovery of the electromagnetic, weak, and strong interactions. Based on fundamental studies, mainly those of Galileo Galilei, Newton^1^ formulated a description of the gravitational interaction in 1687. This physical law, which still gives—to a certain degree—a correct description of many astronomical and terrestrial observations, contains a proportionality factor known as the Newtonian constant of gravitation, abbreviated in equations by the capital letter *G*.

Newton’s theory is correct for table top gravitational experiments in the laboratory but fails to describe the gravitational interaction in strong gravitational fields and at high velocities. For these cases, a relativistic model must be used. General relativity, the current accepted theory of gravitation, was published in 1915 by Einstein^2^ and has been subjected to numerous tests,^3^ all of which it has passed. Gravity emerges in general relativity as a property of space-time. Mass, as well as energy, affects the curvature of space time. The gravitational constant, together with the speed of light, describes the extent to which space-time is contorted for a given mass. This article is concerned only with the limits of gravity where the Newtonian laws are sufficient to describe the system.

*G* was first measured in the laboratory by Henry Cavendish in 1798. Since then, more than 200 experiments have been conducted to precisely determine the value of *G*^4^—with limited success. In June 2015, the Committee on Data for Science and Technology (CODATA) published a new recommendation for the value of *G* with a relative standard uncertainty of 4.7 × 10^−5^. For comparison, with today’s technology, time intervals can be measured to a relative uncertainty of a few parts in 10^18^. The uncertainty of *G* is based on the measurement precision reported by the experimenters, but it also includes a factor of 6.3 in order to take into account the large spread between the values obtained by different groups. This factor reduces the normalized residuals below two, see Sec. VI. The published values scatter by this factor more than they should based on the reported uncertainties. This gives reason to suspect hidden systematic errors in some of the experiments. An alternative explanation is that although the values are reported correctly, some of the reported uncertainties may be lacking significant contributions. The uncertainty budgets can include only what experimenters know and not what they do not know. This missing uncertainty is sometimes referred to as a *dark uncertainty*.^5^ We can assume that each experimenter has provided an uncertainty budget calculation for the experiment that is as detailed as possible, and has excluded possible systematic effects. Even if all experiments of the same type agree, a reasonable way to check for systematic errors is to repeat a measurement with another experimental approach. *G* is in good condition concerning the way in which it is measured. Many different experiments have been conducted and different—sometimes inconsistent—results have been published. The field is definitely far removed from the intellectual phase locking^6^ that has occurred in the past in the context of measuring other fundamental constants of nature.^7^

In this review article, different experimental setups that have been used to determine *G* will be discussed. Where possible, historical background information on precision experiments is provided. Due to the large scope of the topic, details will be largely eschewed in favor of important problems and advances. The list of experiments given here is by no means comprehensive. For a more complete index of measurements, the reader is referred to the work of Gillies,^4^ which was updated in 1997.^8^ For earlier review articles, see Refs. 9–16.

The measurements of *G* have a two-way interaction with the state of the art in technology: (1) Advances in technology can benefit measurements of *G*: for example, the beam balance has become sensitive enough to measure local gravity variations.^17^ (2) The technology developed to measure *G* can have a valuable impact on other areas of technology, such as the torsion balance for geophysical prospecting.^18^ One purpose of this text is to motivate more scientists to measure *G* and to learn from the many interesting experimental techniques deployed in the field. Although the experiment is difficult, it provides an excellent training ground for future scientists and engineers.

This article is organized into seven sections. Section II gives additional background information on the gravitational constant, including its applications. In Sec. III, general facts about the measurement of *G* are described, and different methods are briefly introduced. Section IV describes several different measurement principles in greater detail. In Sec. V, considerations regarding large masses (field masses) are examined. After an outlook in Sec. VI, the main conclusions of the article are discussed.

## II. BACKGROUND

**FIG. 1.**
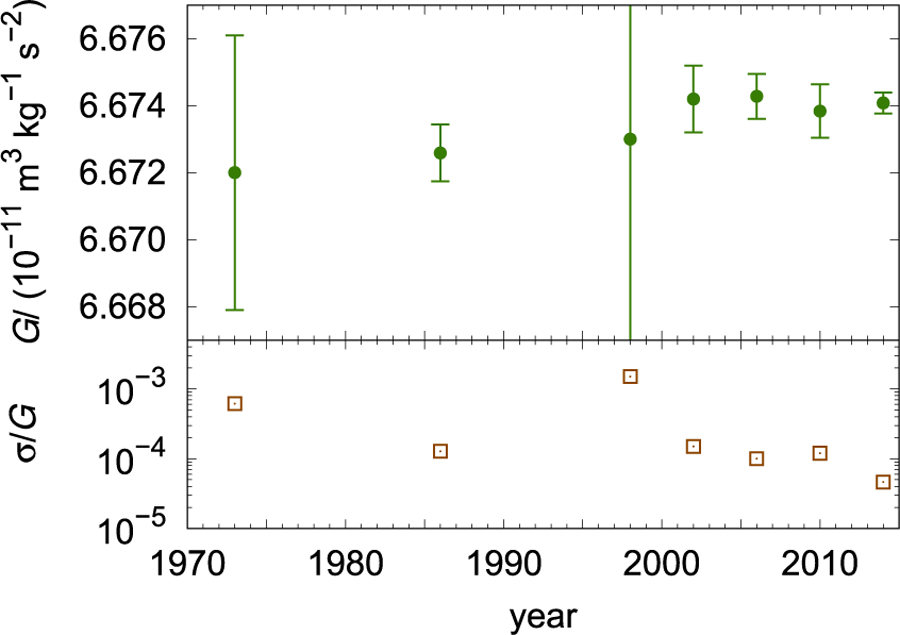
The values of *G* recommended by the Task Group on Fundamental Constants of the Committee on Data for Science and Technology over the past several decades. The lower graph shows the relative standard uncertainty assigned to each recommendation. The values and uncertainties are obtained from Refs. 19 and 21–26.

The measurement of the gravitational constant has a long history. It was the second fundamental physical constant ever measured, preceded only by the speed of light. The numerical value of the speed of light was fixed with zero uncertainty in 1983 in order to define the unit of length, the meter. Of the fundamental constants that can still be measured, *G* has the longest measurement history. The latest CODATA recommendation^19^ assigns a value of 6.674 08 × 10^−11^ m^3^ kg^−1^ s^−2^, with a relative measurement uncertainty of 4.7 × 10^−5^, to *G* (Fig. 1). For comparison, shortly before the fixed value of the speed of light was established, a relative uncertainty of 3.5 × 10^−9 20^ was assigned to its value. At first glance, it seems difficult to understand that—after more than 200 years and over 200 measurements—the assigned relative uncertainty of *G* is as high as it is.

### A. From the mean density of the Earth to *G*

The physical quantity discussed here appears in Newton’s law of gravitation. This law was formulated by Newton in 1687,^1^ and today is often written in the form

(1) $$F=G\frac{m_{1}m_{2}}{r^{2}},$$

where *m*_1_ and *m*_2_ denote the masses of two bodies with a distance *r* between their centers of mass. *G* plays the role of a constant of proportionality (i.e., it can be considered as conversion factor). In fact, when Newton wrote the law of gravitation, he did not introduce this proportionality factor because at that time, laws were formulated as ratios rather than as equations. Hence, *G* was of no significance to Newton. Scientists were more interested in how much a celestial body weighed compared to the Earth or the Sun. Thus, originally, Cavendish did not measure *G* in his famous experiment but the mean density of the Earth, as his article was titled.^27^ He calibrated the density of the field masses with respect to a reference material (water). Then, he measured the attraction of the test masses with respect to these field masses. As a result, he was able to give a ratio between the field masses and the Earth’s mass. As the diameter of the Earth was already known, he was able to derive its mean density.

Although the constant of proportionality was not present in Newton’s original publication, today, his law is written as an equation, in the form (1). It is believed that Siméon Denis Poisson was the first to introduce a constant of proportionality in 1811 (see Ref. 28) although he did not use the character *G*, which is now usually used for this constant. *G* appeared, in all likelihood, for the first time in a publication by König and Richarz in the year 1885.^29^ Currently, *G* is often also called “Big *G*” in order to distinguish it from the acceleration due to gravity, which is denoted by the lower-case letter *g*—hence referred to as “Little *g*.”

### B. Applications

**FIG. 2.**
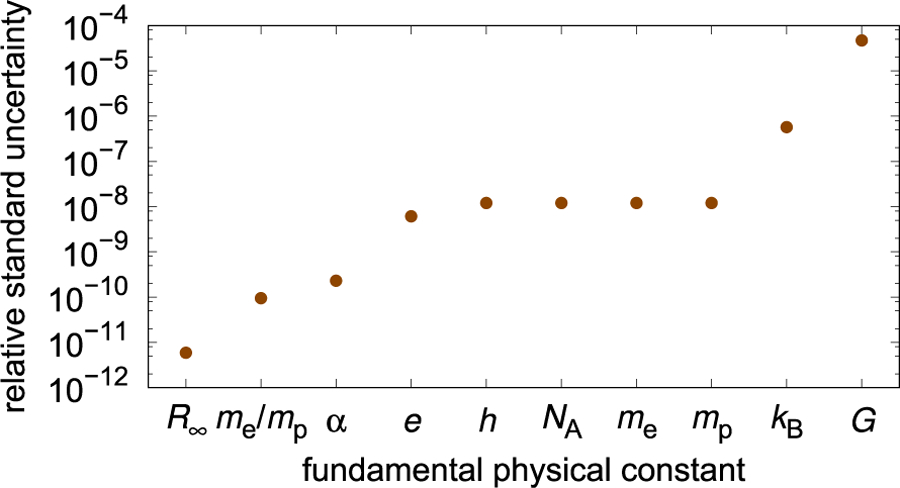
The relative standard uncertainties of the recommended values of selected fundamental constants. The uncertainties are obtained from the latest adjustment of the fundamental constants by the Task Group on Fundamental Constants under the auspices of the Committee on Data for Science and Technology (CODATA). A list of all recommended values can be found in Ref. 19. The uncertainty of *G* is the largest of the known fundamental constants of nature. Note that the constants that are known with the smallest uncertainties are all determined by means of measurements of frequencies or ratios of frequencies.

The current relative standard uncertainty of *G* is 4.7 × 10^−5^, according to the 2014 least squares adjustment performed by CODATA. This is a relatively large uncertainty when compared to the uncertainties of other fundamental constants (see Fig. 2). One may ask why the large relative uncertainty of *G* is not a problem in science or commerce. To answer this question, the fields and applications that require *G* will be briefly discussed.

#### 1. Metrology

Metrology institutes such as Physikalisch-Technische Bundesanstalt (PTB) in Germany and National Institute of Standards and Technology (NIST) in the United States, according to their statutes, have a mandate to measure fundamental constants. The current measurement uncertainty attributed to *G* illustrates the fact that measuring it is a very difficult task although many scientists are drawn to the challenge of reducing its uncertainty.

Another possible application of *G* in metrology can only be realized if its uncertainty is further reduced. In 1899, Max Planck proposed a natural system of units^28,30^ that was not reliant upon artifacts. He proposed using the velocity of light, *c*, Planck’s constant, *h*, and Newton’s constant of gravitation, *G*, to define a fundamental system of units. In this system, the unit of mass—called the Planck mass—is defined as

(2) $$m_{p}=\sqrt{\frac{\hbar c}{G}},$$

where ℏ=h/(2π), with a value of 21.765 *μ*g, when expressed in the International System of Units (SI). This system of units was conceived to be valid everywhere in the universe and for all times. For this system of units to be competitive with the existing SI system, the uncertainties must be equal or better than those of the current SI system. However, in the SI, mass at the highest level can be measured to a few parts in 10^9^, whereas *G* is only known to be 5 parts in 10^5^. The relative uncertainties in mass metrology would increase about 10 000-fold if the Planck units were chosen. In addition to the Planck mass, a Planck length and a Planck time can be established. All three Planck units require *G*. The Planck time and Planck length play important roles in astrophysics and particle physics. In the planned revision of the International System of units, the SI, fundamental constants will be used to define the unit system although *G* will not be used.

#### 2. Fundamental physics

One serious shortcoming of the theory of gravitation is that it cannot be unified with the other three interactions. Many alternative theories of gravitation have been developed, some of which differ only in minor details. Several of these theories predict either a value for *G* (string theory) or variations of *G* in space or time (the Brans-Dicke theory). Some scientists believe that there is a possible slight deviation from Newton’s inverse square law (i.e., the gravitational force is not proportional to 1/*r*^2^). It is assumed that there is a hidden “fifth force,” one which, like gravitation, is proportional to mass, but whose strength changes below a certain separation. More accurate knowledge of *G* can help in eliminating some of these theories and developing entirely new concepts. Furthermore, having an accurate number for *G* can be useful for disproving theories that predict a different number for *G*.

#### 3. Geophysics and astronomy

Using observational methods, the geocentric constant of gravitation, *GM*_Earth_, can be determined with a relative uncertainty of only 2 × 10^−9^ although the value of *M*_Earth_ can be given only with the uncertainty of *G*. The determination of the Earth’s mean density is also limited by the accuracy with which *G* is known. Better knowledge of the mean density of the Earth is still desired in geophysics, as current knowledge limits the calibration accuracy of gravity gradiometers.^31^ Gravity gradiometers with better calibrations lead to better data in geophysical prospecting. McQueen^32^ pointed out that the uncertainty in the Earth’s elasticity parameters (Love numbers) is also limited by the uncertainty in *G*. In astronomy, *G* alone is rarely of importance, as most effects are caused by the product of *GM*, where *M* is the mass of a (central) star. Even the most recent measurement of the mass of a star from the relativistic deflection of star’s light was performed at first by means of *GM*.^33^ Furthermore, star masses are very often given as a ratio to the solar mass, whose measurement is again obtained from a measured product of $GM_{⊙}$.

## III. GENERAL CONSIDERATIONS FOR MEASUREMENTS

In SI units, the quantity *G* has the unit m^3^ s^−2^ kg^−1^. In order to determine *G*, quantities with the dimensions of length, time, and mass have to be measured. In SI units, the numerical value {*G*} = 6.674 08 × 10^−11^ is very small (ten orders below unity). The size of the unitless number indicates that the associated forces between objects in a laboratory are small, in terms of the units used in everyday life. This provides a first indication of why it is so difficult to measure *G* accurately. The gravitational force between two spherical masses of 1 kg at a distance of 1 m is 67 pN, which equals the weight of a mass of about 6.7 ng. For comparison, the mass of a human cell is approximately 1 ng.^34^ From this thought experiment, it is clear that at least two large masses have to be brought as close together as possible in order to maximize the signal. This requires for a high volumetric mass density that allows large masses to be brought close to each other.

### A. Microgravity environment

**FIG. 3.**
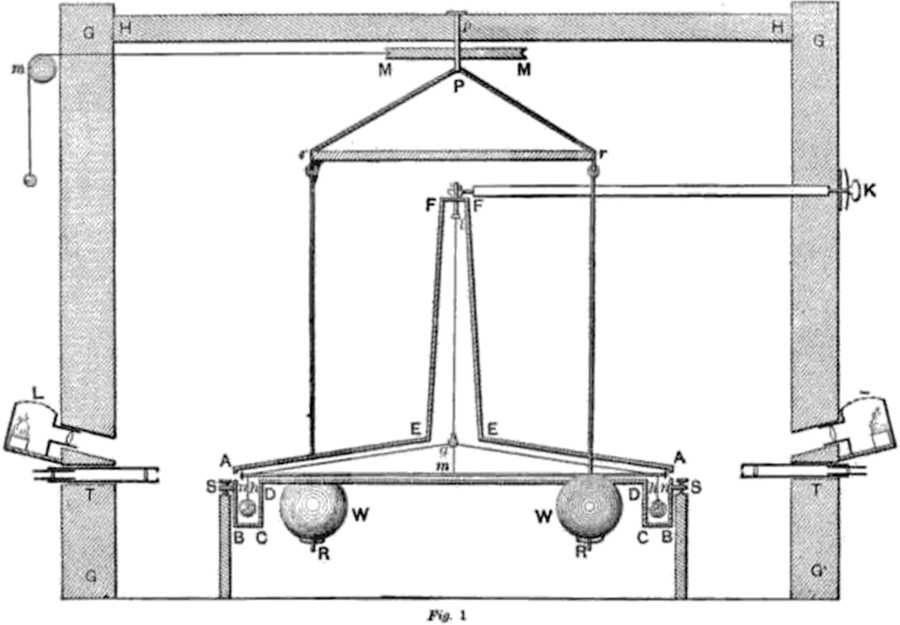
The torsion balance used by Cavendish in the first laboratory measurement of *G*. Adapted from Ref. 12.

Equation (1) indicates two methods of measuring *G*: either by measuring the (attracting) force between two well-characterized masses or by measuring the acceleration of one mass towards the other, *a*, which is

(3) $$a=G\frac{m_{1}}{r^{2}},$$

where *m*_1_ denotes the field (generating) mass. Note that Newton’s law of gravitation is symmetric for the masses *m*_1_ and *m*_2_. However, the terms *field mass* and *test mass* are used to describe these experiments. The test mass is connected to a sensor, while the position of the field mass is usually changed in regular time intervals in order to modulate the gravitational signal. Although the distinction is made here and in most articles describing the experiments, one should be aware of the inherent symmetry of the gravitational law. A force (or more precisely, torque) measurement was used in the first laboratory experiment to determine *G*. In 1798, Henry Cavendish used a torsion balance, shown in Fig. 3, to determine *G* from the gravitational torque acting on a dumbbell suspended from a torsion fiber. The apparatus was designed and built by Rev. John Michell; however, he did not complete the apparatus or perform any experiments on it before his death in 1793. Michell invented the apparatus with the purpose of determining the mean density of the Earth. Rev. F. J. H. Wollaston later forwarded it to Cavendish, who carried out the now-famous measurement.^12^ Independently, Charles A. de Coulomb invented a torsion balance in 1777. Gehler^35^ pointed out that Coulomb was the first to use fibers to suspend a torsion pendulum, experimenting at first with hairs and silk fibers before employing metal fibers at a later time. Michell, on the other hand, began by balancing the torsion bob, a thin wire, on a tip, similar to a compass needle, in 1768. Later, he adopted a fiber to suspend a dumbbell. One noteworthy feature of the torsion balance is that it decouples the vertical gravitational force caused by the Earth from the horizontal one caused by the field masses. This is necessary because if the attractive force between two bodies is measured vertically, the signal will be between a million (10^6^) and a trillion (10^12^) times smaller than the background caused by the gravitational attraction due to the Earth. The exact signal-to-background ratio depends on the size and geometry of the mass assembly.

**FIG. 4.**
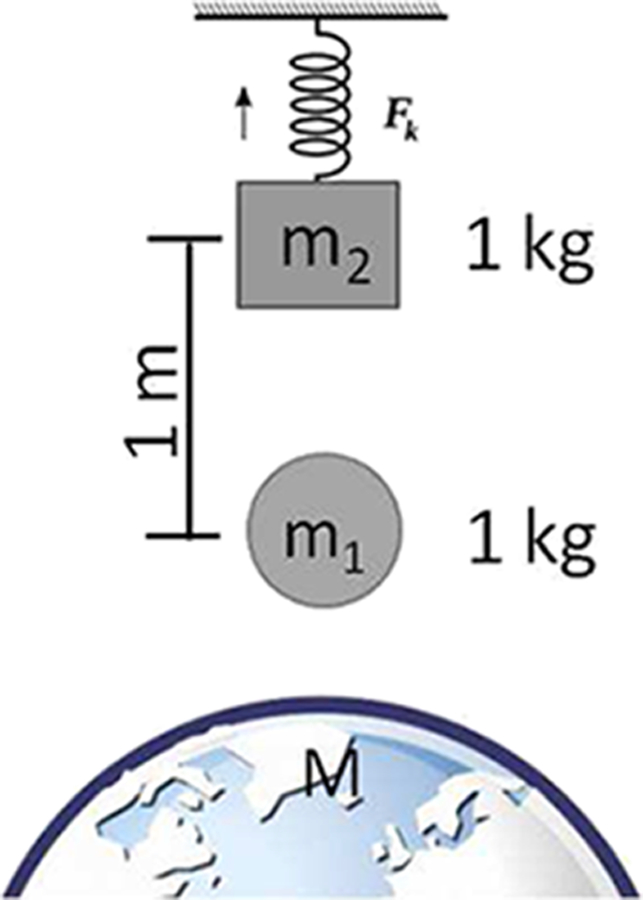
Example of how to measure *G* by means of a spring balance. First, the elongation of the spring due to the Earth’s mass, *M*, is measured. Then, an additional field mass *m*_1_ is added, and the change in elongation is measured. If an elongation of, for example, 10 cm results due to the Earth’s mass, then the field mass results in a variation of only 0.67 pm.

The disadvantageous signal-to-background ratio is illustrated in Fig. 4. A 1 kg mass is suspended from a spring leading to a 10 cm elongation (to cite one example) due to the gravitational force between the Earth and the mass. A 1 kg field mass is then placed 1 m below the suspended mass, adding an additional gravitational force to the setup. This additional force causes an extra elongation of only 0.67 pm, which is about 1 000 000 times smaller than one wavelength of light. This measurement is thus a very challenging one to perform, considering environmental factors that cause changes in the spring constant and, thus, drift in the mass position. To make matters worse, the local gravitational acceleration *g* is not constant, but it is a function of time due to the apparent motion of the Sun and the Moon, both of which exert additional forces known as tides. The relative change of *g* due to the tides has a peak-to-peak amplitude of about 3 × 10^−7^ (i.e., the change in force due to the tides is 15 000 times larger than the force produced by the field mass). The brief example of the spring balance clearly illustrates the merit of the torsion balance. Cavendish’s setup maintains both attracting masses at the same vertical height, and the attracting horizontal force is observed. Apart from the horizontal gravity gradients of the Earth’s gravity field and the surrounding masses in the laboratory, this force contains only the gravitational attraction due to the field and test masses. Since the torsion wire in Cavendish’s setup has a very small restoring torque, small external torques can be measured. We have created a microgravity environment for one degree of freedom, the rotation of the torsion bob about the fiber axis. The lack of (angular) accelerations along this direction allows for long measurement times. Although the torsion wire is a crucial component in this setup, it is also an important source of error, as was shown in Ref. 36, see Sec. IV A 2 b.

A microgravity environment, with accelerations approximately 10^−6^ *g*, can also be achieved in an artificial satellite. The field mass and test mass fall on a circular orbit around the Earth, and the relative acceleration between the two is only given by the small gradient of the gravitational field and the gravitational attraction between the two masses. Unlike a torsion balance, such a system can be realized without the disturbing properties of a suspension—the two masses can float within the satellite without a mechanical connection to the satellite. Such a drag-free test mass was recently successful demonstrated by the LISA (Laser Interferometer Space Antenna) Pathfinder mission.^37^

Observing the mutual gravitational attraction in a microgravity environment entails very long integration times since (in principle) the bodies continue falling forever.

However, space experiments are usually prohibitively expensive and require long preparation times, often over several decades. To date, several ideas for space experiments to determine *G* have been proposed.^38^ Only one such proposal, called “Satellite Energy Exchange (SEE),” is still being actively prepared.^39^

To avoid costly space experiments, other possibilities that allow free-fall are appealing. Obviously, on Earth, very long integration times cannot be achieved, as the object falls towards the Earth and not around it (as it does in space). The drop tower in Bremen, Germany, for example, allows a drop duration of 9.3 s.^40^ In some cases, this may not be long enough to obtain a good measurement of *G*; unfortunately, the facility’s tight schedule prevents many repetitions from taking place in succession (even if this were possible, it would become very expensive over time). Another option is sounding rockets. Experiments on sounding rockets have been considered for testing the equivalence principle, but not for measuring *G*.^41^

### B. Free-fall method

In the example above, the additional field mass between the Earth and the suspended mass alters the local acceleration *g* at the position of the test mass. This idea can and has been used to determine *G*. Free-fall absolute gravimeters are precision instruments that can measure the local acceleration with relative uncertainties on the order of 10^−9^. Gravimeters have a broad application in geodesy and geophysics and have recently been used in fundamental metrology as well for the planned re-definition of the SI unit of the kilogram via the Kibble (or watt) balance.^42^ In contrast to space experiments, however, only one mass is in free-fall. A well-known field mass can be placed close to the trajectory of the test mass. The gravimeter measures the perturbed local gravity. By modeling the perturbation produced by the field mass, *G* can be determined.

### C. Beam balance

Besides the torsion balance and absolute gravimeters, a third instrument can be used to measure *G*: a beam balance. If a mass of, for example, 1 kg is placed on each side of a beam balance, the balance will stay in equilibrium as long as the local gravity at the positions of each mass is equal. If a heavy field mass is placed close to one of the test masses, the local gravity at this test mass will change and the balance will get out of equilibrium since the forces on both masses are different. By measuring the force difference, we can determine *G* if the field and test mass are well characterized. To a certain extent, this method is similar to the torsion balance method: The gravitational pull from the Earth is supported by the fulcrum and only differential forces cause an indication of the balance. Newer versions of precision balances use an electromagnetic force compensation; as a result, the mechanical weight on one side is compensated by an electromagnetic force on the other side. In this case, however, the tidal variations of gravity have to be taken into account.

### D. Simple pendulum

Finally, a simple pendulum can also be used to determine *G*. With a pendulum, there are basically two ways of measuring *G*. The first is to measure the frequency change of the pendulum swing when a field mass is placed below the pendulum bob. This is possible because the swing time, *T*, depends on gravity, *g*, as

(4) $$T^{2}=4\pi^{2}\frac{l}{g},$$

where *l* is the pendulum length. A second way is to deflect the pendulum bob by placing a field mass horizontally next to it. It becomes obvious that both ways are similar to variants of the torsion balance—namely, the time-of-swing method and the simple deflection (Cavendish) method, the difference being that the torsion balance has a higher sensitivity.^43^

## IV. MEASUREMENT PRINCIPLES

In Sec. III, a brief overview of the different approaches to measure *G* was given. In Secs. IV A–IV D, the different techniques are described in more detail.

### A. Torsion balance

**FIG. 5.**
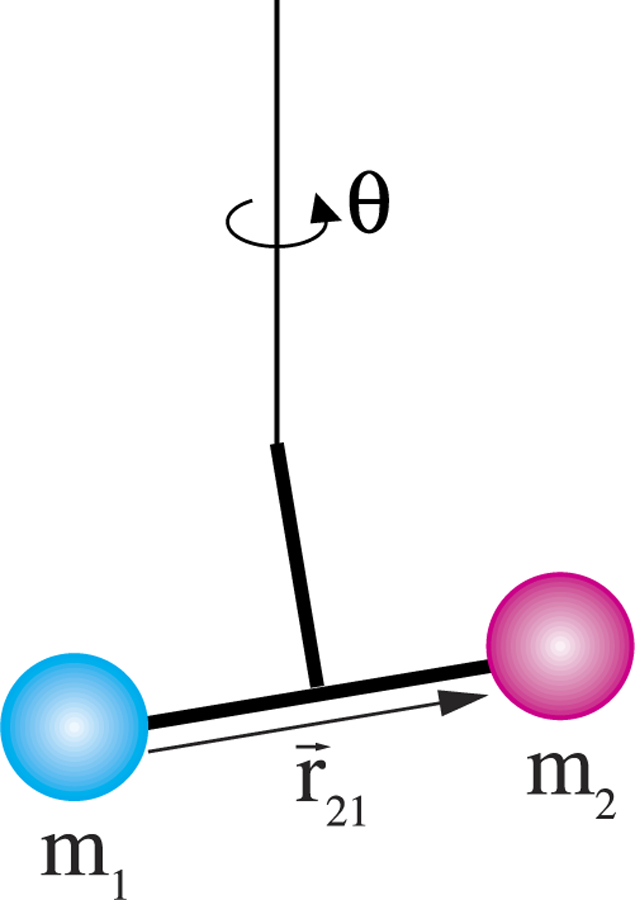
Schematic drawing of a simplified torsion balance. The torsion fiber hangs vertically like a plumb line. The pendulum bob shown here is called the dumbbell. If the torsion balance is not balanced (i.e., $m_{1}\neq m_{2}$), the bob is angled with respect to the horizontal plane such that the center of mass (COM) is below the suspension point. The fiber is only sensitive to torques around its axis, which is vertical.

A torsion pendulum is a standard example of a simple harmonic oscillator. It consists of two components: the torsion fiber, which provides a restoring torque, and the pendulum bob, which contributes inertia (see Fig. 5). For a freely swinging torsion pendulum, the energy of the pendulum is stored as potential energy in the fiber or as kinetic energy in the rotating bob. The energy switches between these two types of energy every quarter period.

The design of the torsion bob depends on the measurement goal of the torsion balance. Its shape is optimized to allow it to be coupled to external forces that are often modulated. Torsion balances have many applications other than the determination of *G*. A review of torsion balances can be found in Ref. 44.

To measure the influence of the external force on the torsion pendulum, a readout is required. Very often, the readout is performed optically with an autocollimator. In an autocollimator, the divergent beams of a light source are collimated into a parallel beam through a lens. The beam is reflected by a mirror mounted on the torsion pendulum and returned to the same lens. The returning beam is focused onto a position-sensitive detector. By using the same lens twice (for collimation and focusing), the system is not only simpler but also more robust regarding optical aberrations. Further details are available in Refs. 45–47.

#### 1. Basic equations

The differential equation of a torsion pendulum is

(5) $$I\ddot{\theta }+\kappa \left(1+\phi i\right)\theta =N\left(t\right),$$

where *I* is the moment of inertia of the pendulum body, *κ* is the torsional spring constant, *θ* is the azimuthal angle, and *N*(*t*) is the external torque applied at the pendulum bob. The torsion fiber is considered lossy, and *ϕ* denotes the loss angle of the torsion spring, which is the inverse of the quality factor, *ϕ* = 1/*Q*. The damping described by an imaginary component of the torsion constant is called internal damping. Another possibility for damping is velocity-dependent damping, often called external damping. External damping requires an additional term of $\gamma \dot{\theta }$ on the left-hand side of Eq. (5). Gas pressure damping is an example of external damping. Modern torsion balance experiments are (mostly) limited by internal damping, while the external damping term can be ignored. For experiments at room temperature and vacuum using metal fibers, quality factors of several thousands are typical. Hence, the loss angles are very small and the equations can be expanded in a Taylor series around *ϕ* = 0. In the formula below, we expand up to second order.

Without external torques, the torsional angle with starting conditions *θ*(0) = *θ_o_* and $\dot{\theta }\left(0\right)=0$ follows a damped sine according to

(6) $$\theta \left(t\right)=\theta_{o}e^{-t/\tau }e^{i\omega_{o}\left(1+\frac{\phi^{2}}{8}\right)t}\;\;\text{with}\;\omega_{o}=\sqrt{\frac{\kappa }{I}}.$$

The decay time *τ* is given by

(7) $$\tau =\frac{2}{\omega_{o}\phi }=\frac{Q}{\pi f_{o}}\;\;\text{with}\;\omega_{o}=2\pi f_{o}.$$

If the external torque is sinusoidal with angular frequency, i.e., $N\left(t\right)=N_{o}\exp\left(-i\omega t\right)$, the motion of the pendulum at this frequency is given by $\theta \left(t\right)=\theta_{a}\exp\left(-\omega t\right)$ with

(8) $$\frac{\theta_{a}}{N_{o}}=\frac{1}{\kappa }\frac{1-\omega^{2}/\omega_{o}^{2}-i\phi }{{\left(1-\omega^{2}/\omega_{o}^{2}\right)}^{2}+\phi^{2}}.$$

The response to a torque that is more complicated than a pure spectral note can be calculated from the response to each Fourier component by synthesizing the responses.

**FIG. 6.**
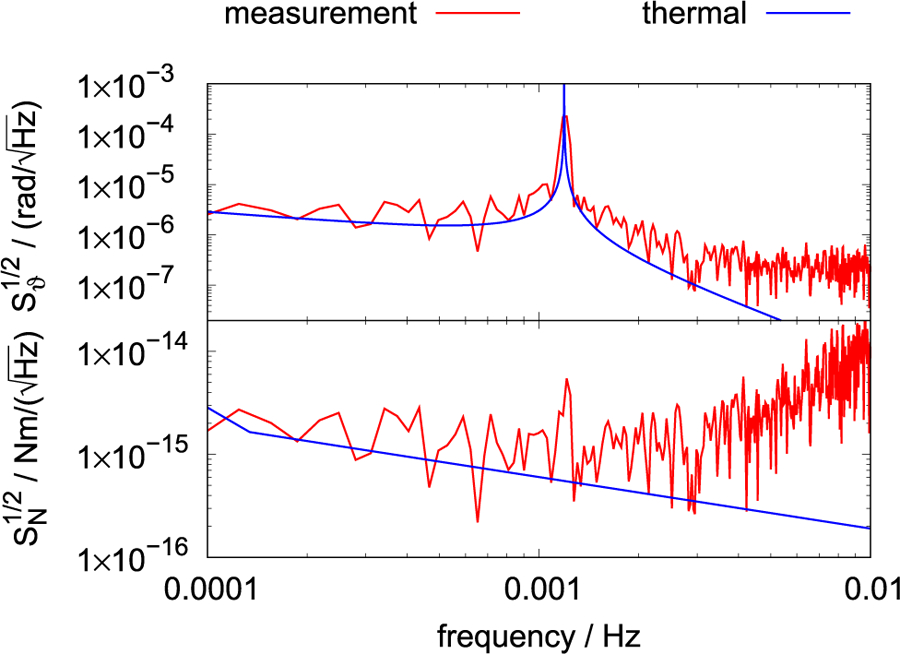
Measured noise and thermal noise of a torsional oscillator with *κ* = 774 pN m rad^−1^. The top plot shows the amplitude spectral density of the torsional angle *θ*. The bottom plot shows the amplitude spectral density of the torque. Ideally, the signal that is to be measured is placed at the minimum value of the torque noise, here about 3 mHz.

The non-negligible loss angle of the fiber has another important experimental consequence: A loss in a system is connected via the fluctuation dissipation theorem to the noise in the system. The single-sided power spectral density *S_τ_*(*f*) of the torque is given by

(9) $$S_{\tau }\left(f\right)\text{d}f=4k_{B}TR\text{d}f,$$

where *k_B_* is the Boltzmann constant, *T* is the temperature of the system, and *R* is the real part of the mechanical impedance,^48^ in this case *R* = *κ*/(2*πQf*). From the spectral density and the known signal frequency, the time that is required to measure a signal with given statistical uncertainty can be calculated. In Fig. 6, the power spectral amplitude of the torque and the angle readout of a torsion balance is shown.

#### 2. Special fibers

According to Eq. (9), the signal-to-noise ratio can be improved by minimizing the term *κ*/*Q* (i.e., the ratio of the imaginary part of the spring constant to the real part). As described in Sec. IV A 2 b, a large quality factor will minimize a systematic bias in the time-of-swing method. Motivated by these two benefits, researchers in recent years have attempted to increase the quality factors of the torsion fibers in their experiments.

Because tungsten has a high tensile strength (and thus, a small restoring constant), tungsten fibers were used in many torsion balances. The quality factor of a torsion pendulum supported by a tungsten fiber can reach values of up to several thousand (see Table I). In order to obtain higher quality factors, different approaches are needed. To date, three different approaches have been used: fused silica fibers, metal fibers at cryogenic temperatures, and torsion strips.

The ultimate yield strength of fused silica fibers is 1000 N m^−2^ or higher.^60,61^ By way of comparison, the yield strengths of tungsten fibers are typically above 2000 N m^−2^. Hence, silica fibers must have a 40 % larger radius to carry the same load as tungsten fibers. A larger radius will increase the torsional stiffness of the fiber, which scales with the fourth power of the radius. Despite this increase in *κ*, fused silica is still a good option since *κ*/*Q* decreases due to its large quality factor. In fused silica, the quality factor is dependent on temperature and frequency. At high frequencies of several thousands of hertz, quality factors of 10^8^ can be achieved. At frequencies typical for torsion balances (from one to several hundreds of mHz), quality factors of 10^6^ are still possible. Unfortunately, because fused silica is an isolator, a pendulum bob suspended by a fused silica fiber is not electrically grounded. This causes increased noise or even systematic effects due to spurious electrostatic forces created by charges on the bob. This problem can be solved with three approaches: discharging the pendulum bob with ultraviolet light,^62,63^ coating the silica fiber with a conductive film,^64^ or sufficient separation between the pendulum body and its surrounding.

Another way in which the thermal noise can be reduced is by lowering the temperature, as doing so involves two beneficial mechanisms. First, the thermal energy *k_b_T* is lowered, and second, the quality factor increases for most metals with decreasing temperature. For fused silica fibers, the dependence of the quality factor on the temperature is more complicated. A detailed investigation^65^ of fibers made from Aluminum 6061 and beryllium copper contains strong evidence of a stick-slip mechanism. A spring with a stick-slip mechanism can be imaged as a series of blocks on a surface connected by springs.^66^

Another interesting suspension for a torsion bob is the torsion strip, a fiber with a rectangular cross section. The use of the torsion strip was pioneered by the group that worked at the BIPM (Bureau International des Poids et Mesures) under the leadership of Terry Quinn. For a rectangular strip with length *L*, width *b*, and thickness *t*, the spring constant is given by

(10) $$\kappa =\frac{bt^{3}F}{3L}+\frac{Mgb^{2}}{12L},$$

where *F* is the material’s shear modulus and *Mg* is the weight of the pendulum bob.^67^ Two terms contribute to the restoring torque. The first term in Eq. (10) is analogous to the elastic term that provides the restoring torque in a circular fiber. The second term is a consequence of the fact that, as the ribbon is twisted, the pendulum bob is raised in the gravitational field of the Earth. Since the gravitational force is conservative, the second term is lossless. The imaginary part of the spring constant is given by the imaginary part of the elastic spring, but the real part of the spring is given by the sum of the elastic spring and the gravitational spring. Hence, the ratio of the imaginary part to the real part is smaller for both components than for the elastic part alone. This mechanism allows for an increase in *Q*. For example, for the strip used in the BIPM experiment, gravity provides about 90 % of the restoring torque. Quality factors on the order of 100 000 were achieved with the BIPM torsion strip.

Table I gives an overview of the properties of the fibers used in recent torsion balance experiments to measure *G*. It can be seen that the torsion strip has a torsion constant that is four to five orders of magnitude larger than that of traditional round fibers. However, since the quality factor of the torsion strip is about one to two orders of magnitude larger than the quality factors of metal fibers at room temperature, the torque sensitivity of the strip is only reduced by a factor of one hundred. This reduced torque sensitivity is compensated by the fact that the torsion strip can carry a greater load; thus, the gravitational torque can be made larger by several orders of magnitude. Finally, a precise experiment can be built using a torsion strip. Note that this conclusion differs from that reached by Boys in 1889,^68^ where he argues in favor of thinnest possible fiber. In the BIPM experiment, the gravitational torque, at 3 × 10^−8^ N m is several orders of magnitude larger than in other torsion balance experiments. The corresponding angular deflection of the pendulum bob is about 30 arc sec.

##### a. Static deflection.

The static deflection method was used by Cavendish to measure the mean density of the Earth. A static torque on a torsion balance causes a deflection from the equilibrium position. The equilibrium position is usually not known, and two measurements are required. The two measurements are performed with two different field mass arrangements. The field masses can be “near” or “far,” or they can be located clockwise or counterclockwise from the torsion fiber. The gravitational torques produced by the field masses on the test masses are denoted *N*_n_ and *N*_f_ for the two field mass positions. The angular excursions of the pendulum bob are given by

(11) $$\kappa \left(\theta_{\text{n}}-\theta_{o}\left(t_{\text{n}}\right)\right)=N_{\text{n}}$$

and

(12) $$\kappa \left(\theta_{\text{f}}-\theta_{o}\left(t_{\text{f}}\right)\right)=N_{\text{f}}.$$

(13) $$\text{Hence},\;\text{if}\;\theta_{o}\left(t_{\text{n}}\right)\approx \theta_{o}\left(t_{\text{f}}\right),\;\text{then}\;\kappa \left(\theta_{\text{n}}-\theta_{\text{f}}\right)\approx N_{\text{n}}-N_{\text{f}}.$$

Here, *θ_o_* denotes the unknown equilibrium position of the pendulum. In general, the equilibrium position is a function of time [i.e., *θ_o_* = *θ_o_*(*t*) due to a slow unwinding of the fiber, usually referred to as drift]. The usual data analysis techniques can be used to suppress this drift.^69–71^ The torque depends on the gravitational constant and a constant that can be calculated from the known mass distribution (i.e., *N_n_* = *Gc_n_*, where *c_n_* has units kg^2^ m^−1^). The unknown torsional stiffness *κ* is obtained from another measurement. The field masses are usually removed or placed in the far position. A measurement of pendulum’s period *T_o_* and a calculation of the moment of inertia *I* yields

(14) $$\kappa =4\pi^{2}\frac{I}{T_{o}^{2}}.$$

In summary, the value of the gravitational constant measured by the static-deflection method is obtained by

(15) $$G=\frac{4\pi^{2}I}{T_{o}^{2}}\frac{\theta_{\text{n}}-\theta_{\text{f}}}{c_{\text{n}}-c_{\text{f}}}.$$

The measurement procedure outlined above relies on several assumptions that are worth considering in more detail:

1. The equilibrium position of the balance *θ_o_* is usually a function of time. As discussed above, the torsion fibers are most realistically modeled as springs with an imaginary spring constant (loss). This thermal noise results in a 1/*f* behavior of the equilibrium position. Hence, the measurements of the two equilibrium positions should be measured within a short time. However, another problem arises: When the torque on the torsional oscillator is changed, it incurs an amplitude. For example, if the torque on the pendulum is abruptly changed by *N*_2_ − *N*_1_, the amplitude gain of the pendulum will be (*N*_2_ − *N*_1_)/*κ*. It is difficult to measure the exact equilibrium position of the pendulum if an oscillation with a large amplitude is present. Several strategies are available to cope with this problem: (a) A torsion pendulum with high damping is used. This eliminates the excitation problem but introduces thermal noise, thus making it difficult to measure the equilibrium position in a feasible time scale. (b) The experiment utilizes switchable damping. The damping is turned on during and shortly after the move and turned off for the measurement of the equilibrium position. Damping can be achieved, for example, by means of active electrostatic feedback. (c) The motion is carefully measured, and the equilibrium position is obtained from a fit of a decaying sine function to the measurements. (d) The motion of the field mass is not abrupt but is optimized so as not to excite the pendulum. One possible motion changes the torque by half and half a pendulum period later by the remaining half. This motion is sometimes referred to as the “crane operator trick.”
2. The torsional constant is measured at the oscillation frequency of the pendulum but is applied to the calculation at (almost) zero frequency. However, the spring constant is due to elasticity that is generally a function of the frequency and *κ*(0) < *κ*(*f_o_*). This problem will be discussed in greater detail in the time-of-swing method. If the effect of the anelasticity is not taken into account, the measured value of *G* will be higher than the true value of *G.*

In modern times, the static mode is hardly used. Notable exceptions are two experiments at the BIPM by Quinn and co-workers.^55,72^ The group built a single torsion balance that could be operated with three different methods: static deflection, time-of-swing, and electrostatic feedback. In the end, two methods—static deflection and electrostatic feedback—were used to determine *G*. The first result was published in 2001. Subsequently, the apparatus was completely rebuilt, and the second result was published in 2013. Both results are consistent with each other. Apart from these two experiments, no other precision experiment has used the static deflection in the past thirty years.

##### b. Time of swing.

In the time-of-swing method, the pendulum’s period is measured with the field masses in two different positions. Similar to the static-deflection method, the field masses are nearby, while for the other mode, the field masses are far away. The gravitational potential adds to the potential of the fiber, thereby increasing the restoring torque. Equation (5) must be modified to add the contribution of the field masses in the “near” and “far” positions to the differential equation. This yields

(16) $$“\text{near}”\;\text{FMs}:I\ddot{\theta }+\left(\kappa +\kappa_{\text{n}}+\kappa \phi i\right)\theta =N_{\text{n}}$$

and

(17) $$“\text{far}”\;\text{FMs}:I\ddot{\theta }+\left(\kappa +\kappa_{\text{f}}+\kappa \phi i\right)\theta =N_{\text{f}}.$$

The spring associated with the gravitational potential is lossless, so the imaginary spring constant in the equations above is derived exclusively from the torsion fiber. Usually, the field masses are placed in such a way that the gravitational torque on the pendulum vanishes, *N*_n_ = *N*_f_ = 0; hence, the equilibrium position of the pendulum remains unchanged between the two states—a notable difference to the static-deflection method. The complex solutions of the angular frequencies squared of Eqs. (16) and (17) are

(18) $$\omega_{\text{n}}^{2}=\frac{\kappa +\kappa_{\text{n}}}{I}+i\frac{\kappa \phi }{I}$$

and

(19) $$\omega_{\text{f}}^{2}=\frac{\kappa +\kappa_{\text{f}}}{I}+i\frac{\kappa \phi }{I}.$$

Hence,

(20) $$\omega_{\text{n}}^{2}-\omega_{\text{f}}^{2}=\frac{\kappa_{\text{n}}-\kappa_{\text{f}}}{I}.$$

The gravitational spring constant depends on the mass distribution and *G* (i.e., *κ*_n,f_ = *k*_n,f_*G*), where, again, the constants have dimensions of kg^2^ m^−1^. Note that the constants *k*_n,f_ are the derivatives of the constants *c*_n,f_ used in the static deflection mode. In summary,

(21) $$G=I\frac{\omega_{\text{n}}^{2}-\omega_{\text{f}}^{2}}{k_{\text{n}}-k_{\text{f}}}.$$

The main advantage of the time-of-swing method is that the primary measurand is a time interval. Besides the metrology of the mass distribution, which is common to all *G* determinations, only periods need to be measured. The time interval is the physical quantity that can be measured with the highest precision, especially in the age of GPS (Global Positioning System). In contrast to the time-of-swing method, the other three torsion-balance methods require the measurement of an absolute quantity, angle, angular acceleration, or feedback voltage. It is much more difficult to measure these quantities with a relative uncertainty of 10^−5^ than it is to measure a time interval with the same relative uncertainty.

The simplicity of measuring a simple time interval has convinced many experimenters in the past thirty years to use the time-of-swing method to measure *G*. In 1982, Luther and Towler published a result with a relative standard uncertainty of 64 × 10^−6^.^49,50^ For many years, this result served as a kind of “gold standard” among *G* experiments. Fourteen years later, Karagioz and Izmailov published a new result using the time-of-swing method. The main purpose of their experiment was to search for violation of the inverse square law by measuring *G* with field masses at several distances. Since then, they have continued to collect data with their apparatus. In 1997, Luther published another result with the doctoral student Bagley.^52^ The catalyst for this work was the discovery of the Kuroda effect (see below). The first measurement by the gravity group at the Huazhong University of Science and Technology (HUST) under the leadership of Luo was published in 1998.^73,74^ The group published a second result in 2010 with a relative standard uncertainty of 26 × 10^−6^.^57^ More recently, Newman and his collaborators published their results using a cryogenic torsion pendulum. Riley’s team achieved a relative standard uncertainty of 19 × 10^−6^.^59^

The time-of-swing method appeared in print in an article by Reich^75^ in 1852. Reich attributes the idea to Forbes (see Ref. 11). However, Mackenzie^12^ mentions that this method was first proposed by Muncke in 1827^76^ and that the mathematical analysis was given by Brandes in 1806.^77^ Bouguer’s pendulum measurements during his Peru expedition, on the other hand, can be considered the very first time-of-swing experiment (see Sec. IV B).

Forty-four years after Reich’s publication, Braun started experiments that were reported in a detailed publication in 1896.^78^ Since then, the time-of-swing method has gained popularity with other researchers because of its simplicity. It took over one hundred years before a serious problem with the time-of-swing method was discovered by Kuroda:^36^ The implicit assumption of the time-of-swing method is that the torsional constant of the fiber is the same for both states of the field masses. Then, *κ* cancels from Eqs. (16) and (17). However, this assumption cannot hold perfectly since the torsional oscillator swings at different frequencies and the presence of an imaginary spring constant requires a frequency-dependent real part due to the Kramers-Kronig relations.

**FIG. 7.**
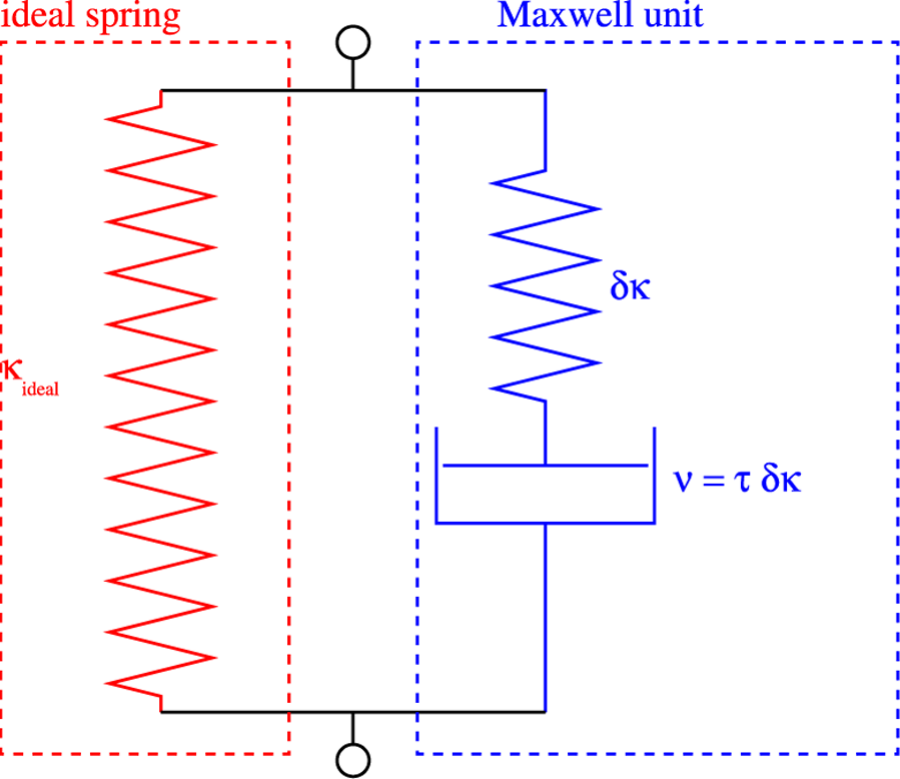
A simple model of a real spring.

**FIG. 8.**
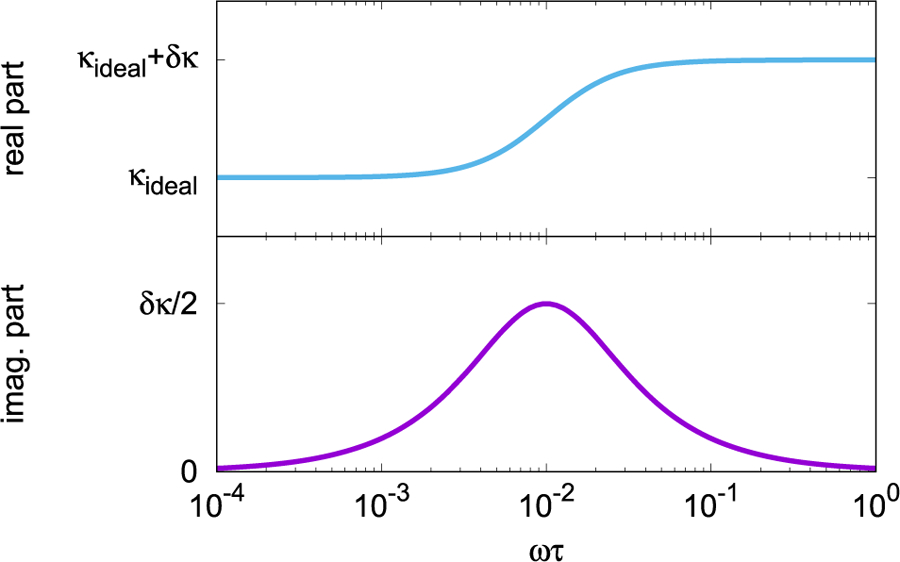
The real and imaginary parts of a simple model of a real spring consisting of an ideal spring in parallel to a Maxwell unit (see Fig. 7).

The theoretical frequency of the spring constant depends on the model that is used for the spring. The simplest model for a spring is a parallel circuit (Fig. 7) of an ideal spring and a Maxwell unit. A Maxwell unit is the serial connection of a spring and a dissipative element (dashpot). The ideal spring is characterized by its spring constant *κ*_ideal_. The Maxwell unit can be characterized by the spring constant *δκ* of its spring and a time constant *τ*. If a sinusoidal force is applied to the simplified model, the total spring constant depends on the angular frequency of the force, *κ*(*ω*). For $\omega \ll \tau^{-1}$, the resulting spring constant is $\kappa_{\text{res}}\approx \kappa_{\text{ideal}}$; for $\omega \gg \tau^{-1}$, it is $\kappa_{\text{res}}\approx \kappa_{\text{ideal}}+\delta \kappa$ (see Fig. 8).

Figure 8 also shows the imaginary part of the spring constant, which is a measure of the loss in the system. For the simplified model discussed here, the imaginary spring constant reaches a maximum of *δκ*/2 at an angular frequency of ω = *τ*^−1^. For this model, the maximum of the imaginary part of the spring constant is exactly half the change in the real part of the spring constant. Hence, one way to find a material that has a small dependence of the spring constant on the oscillation frequency is to search for a material with small loss or large *Q*.

Clearly, a dependence of *κ* on *ω* is undesirable, but how does it influence a *G* measurement using the time-of-swing method? For the *G* experiment, the higher oscillation frequency is in the “near” position with the masses (i.e., in line with the pendulum at the equilibrium position). In this geometry, the gravitational torque adds to the restoring torque of the fiber, yielding an increased torsional frequency. Hence, Eq. (20) needs to be amended to read

(22) $$\omega_{\text{n}}^{2}-\omega_{\text{f}}^{2}=\frac{\kappa_{\text{n}}-\kappa_{\text{f}}+\kappa \left(\omega_{\text{n}}\right)-\kappa \left(\omega_{\text{f}}\right)}{I},$$

where $\kappa \left(\omega_{\text{n}}\right)-\kappa \left(\omega_{\text{f}}\right)>0$. The Newtonian constant is now obtained from

(23) $$G=I\frac{\omega_{\text{n}}^{2}-\omega_{\text{f}}^{2}}{k_{\text{n}}-k_{\text{f}}}-\frac{\kappa \left(\omega_{\text{n}}\right)-\kappa \left(\omega_{\text{f}}\right)}{k_{\text{n}}-k_{\text{f}}}.$$

From the measured frequency difference, a small positive correction term must be subtracted to obtain the value of *G*. If the experimenter “forgets” to subtract this correction, the reported value of *G* will be too high.

The model with a single Maxwell unit is very simple and does not describe a real spring. However, the qualitative conclusions drawn from the simple model remain valid. More realistic models assume a distribution of Maxwell units that are in parallel to an ideal spring. The distribution covers a continuum in values of the time constant and the oscillator strength *δκ*.

Depending on the model used for the distribution of the Maxwell units, several limits of the bias for the *G* measurements can be estimated. Kuroda^36^ estimated that the relative bias in the *G* measurement is <*Q*^−1^/*π* (i.e., without correction, the result would be relatively higher by this amount). Kuroda’s estimate rests on the assumption that the ratio of the imaginary and the real parts of the torsion constant is fixed. Newman^79^ set a different limit of <*Q*^−1^/2 using a continuous distribution of Maxwell units.

This effect is, in addition to the thermal noise arguments discussed above, another reason to use special fibers with high *Q* (see Sec. IV A). In recent years, several experiments have been carried out using the time-of-swing method. Table II summarizes the key parameters.

An interesting and well-documented time-of-swing measurement was carried out by Newman *et al.*,^59,65,79^ who built a cryogenic torsion pendulum to increase the quality factor and thereby decrease the bias introduced by the Kuroda effect. The fiber was suspended from a stage at 2.5 K. At the other end of the fiber, a flat plate was mounted. As is described in Sec. IV A 2 d, the exact dimensions are less crucial, simplifying the test mass metrology. Two field masses shaped like doughnuts were used to generate a very uniform gravitational field, see Sec. V. Since the field masses were at room temperature and the plate was at cryogenic temperatures, the Dewar had to be between the field masses and the pendulum, resulting in a large distance between the two. For this reason, the gravitational signal is very small, about three orders of magnitude smaller than in other time-of-swing methods (see Table II). Another interesting feature of this experiment is that the measurement is performed at various oscillation amplitudes up to slightly more than one complete revolution. Three different fibers were used (see Table I), and detailed studies of each fiber material were carried out. The relative uncertainty of the final result is 19 × 10^−6^.

##### c. Torque feedback.

In general, one successful strategy used to measure an unknown quantity is to compensate the effect of the unknown on a system by means of an effect that is known or calculable. Specifically, the gravitational torque of a modulated mass arrangement can be compensated by an electrostatic torque. The torsion balance acts as a null detector, while the measured torsion angle is used as the input signal for a control loop that adjusts the voltages on the electrodes to zero the input. Since no torsional excursion occurs, the measurement is not affected by the Kuroda effect discussed in Sec. IV A 2 b. Nevertheless, it is beneficial to employ a fiber with a large quality factor because the power spectral density of the torque cannot be lower than the expression given in Eq. (9).

Evidence suggests that Dicke was the first to operate a torsion balance with electrostatic feedback^80^ in order to test the equivalence principle in 1964. In 1980, de Boer *et al.* from PTB proposed a setup with electrostatic feedback to measure *G*.^81^ A first result was published in 1987.^82^ Recently, two groups have used this method to operate a torsion balance: Armstrong and Fitzgerald at the Measurement Standard Laboratory in New Zealand^56,83–85^ and a group under the leadership of Quinn at the BIPM in France. The group at the BIPM published two results with two different torsion balances. One result was published in 2001^55^ and the other was published in 2013.^72^ A complete overview of both experiments can be found in Ref. 86.

An argument frequently made is that the electrostatic interaction is many orders of magnitude larger than the gravitational interaction. For example, the electrostatic force between a proton and an electron in the hydrogen atom is 10^38^ times larger than the gravitational force between the two. One could conclude that there is no way to control the electrostatic feedback well enough to measure the effect of the gravitational force. However, this frequently made comparison is flawed because of the huge charge-to-mass ratio of the electron and the proton. Of the two, the proton has the smaller charge-to-mass ratio, which is 10^8^ C kg^−1^. By way of comparison, the charge-to-mass ratio of an aluminum ball with a mass of 5 g and a potential of 1 V (typical numbers for an object in a laboratory) is 18 orders of magnitude smaller. With the latter number, and by increasing the distance of the charge, the electrostatic feedback seems more realistic.

The electrostatic energy on a capacitor with capacitance *C* at voltage *V* is

(24) $$W=\frac{1}{2}CV^{2}.$$

If one of the electrodes is connected to the torsion balance, the capacitance (and thus, the electrostatic energy) is a function of the torsional angle *θ* of the pendulum bob. If the system is not at the angle where the system has minimal energy, a torque toward this direction arises,

(25) $$N=-\frac{1}{2}\frac{\text{d}C}{\text{d}\theta }V^{2}.$$

The source masses are modulated between two states, producing two torques, *N*_n_ = *Gc*_n_ and *N*_f_ = *Gc*_f_, where *c*_n,f_ denote constants that are calculated from the mass arrangement of the experiment. For each of the two states, the torques are balanced using the voltages *V*_n_ and *V*_f_, which can be measured with a precision voltmeter. From the difference, *G* can be obtained as follows:

(26) $$G=\frac{1}{2}\frac{\text{d}C}{\text{d}\theta }\frac{V_{\text{f}}^{2}-V_{\text{n}}^{2}}{c_{\text{f}}-c_{\text{n}}}.$$

The capacitance gradient, d*C*/d*θ*, is measured in a separate measurement. One strategy used to measure the capacitance gradient is to excite the pendulum to a large torsional motion and to measure the capacitance with a (commercial) capacitance bridge and the angle with an autocollimator. A numerical derivative is then calculated from these measurements.

Several considerations are important for the operation of a torsion balance in the electrostatic feedback mode:

###### Electrostatic forces are unidirectional:

With one set of electrodes, only a unidirectional torque can be generated. Hence, either two sets of electrodes must be used or the feedback position must be offset from the equilibrium position of the torsional oscillator in such a way that the fiber provides the torque in the other direction.

###### AC versus DC:

Capacitance measurements are usually carried out at audio frequencies. The bridge shown in Ref. 87 is a typical example of a circuit that is used for this measurement. The voltage applied to the electrode can either be alternating current (AC) or direct current (DC). The former is more difficult to measure precisely; precise measurements of AC voltages are usually made using thermal converters that find an equivalent DC voltage, which is eventually traceable to the Josephson effect. For the latter, the frequency dependence of the capacitance must be well understood.

###### Contact potentials:

Applying a voltage to the metal does not always result in the same potential at the electrode surface due to surface and contact potentials, which can be as large as several hundreds of mV. This problem can be solved by reversing the voltage. In one case, the torque is proportional to (*V*_s_ − *V*_n_)^2^, and in the other case, it is proportional to (*V*_s_ + *V*_p_)^2^. Here *V*_p_ and *V*_n_ are both positive values. If the torques produced are nominally the same, the surface voltage can be obtained, *V*_s_ = (*V*_n_ − *V*_p_)/2.

###### Parasitic capacitances:

Equation (24) is an approximation if there are only two conducting surfaces and the potential difference between the two surfaces is *V*. In reality, it is extremely difficult to achieve an electrostatic setup, where only two surfaces matter. If there are more than two surfaces, Eq. (24) has to be a sum over all combinations of electrode pairs. Each summand contains the cross capacitance and the squared voltage differences.^88,89^ All cross capacitances that depend on the angle of the torsion balance will contribute to the electrostatic torque. Notably, one such component was mistakenly left out by a *G* experiment.^90,91^ This led to a large systematic bias and eventually to the withdrawal of the result.

Electrostatic force measurements are becoming more important due to the impending revision of the International System of Units (SI). In the revised SI, the unit of mass is no longer given via an artifact but can be realized from a fixed value of Planck’s constant, *h*. For small mass values, electrostatic balances can be used to realize the unit of mass.^92^ Here, “Big *G*” experiments and new technical demands can benefit from each other. Both fields require a solid understanding of the absolute magnitude of electrostatic torques and forces.

The electrostatic feedback method, as well as the static deflection method, relies on an absolute calibrated angle readout which is necessary to calculate the capacitance gradient. Quinn *et al.* found an interesting way to take advantage of the fact that *θ* appears in the denominator in Eq. (26) and in the numerator in Eq. (15). By combining the two methods in one apparatus and averaging the results, the uncertainties due to the angle calibration are anti-correlated.^86^ This negative correlation substantially reduces the effect of the calibration of the autocollimator on the average value. For example, in the 2010 measurement performed by the BIPM group, the relative uncertainty of the angle calibration is 47 × 10^−6^. The total relative standard uncertainty of the average of both the measurements is only 25 × 10^−6^, which is achieved by combining the two anti-correlated measurements.

##### d. Angular-acceleration feedback.

According to available information, the angular-acceleration method was first discussed by Rose *et al.*^93^ in 1969 and was perfected by Gundlach^53^ in 2000. For this method, the torsion balance is mounted on a turntable. The principal idea is that the gravitational torque acting on the pendulum is compensated by an inertial torque: The gravitational torques between the source masses and the pendulum bob produce an angular acceleration of the pendulum bob in the direction of the position with the lowest potential energy. A feedback-control loop accelerates the turntable in such a way that the torsion bob does not move with respect to the rotating reference frame. The data to obtain *G* are extracted from the angular acceleration of the turntable, which (at least for the infinite gain of the control loop) is identical to the gravitational angular acceleration acting on the pendulum bob.

The angular-acceleration feedback method has three advantages: (1) The fiber does not twist since the pendulum remains stationary in the rotating reference frame; thus, the experiment is not affected by the Kuroda effect. (2) Measuring the acceleration has the advantage that the mass of the pendulum bob (and even some of its dimensions) is canceled out (see below). In brief, the cancellation is analogous to the cancellation of the mass of a dropping object in a gravitational field: From *mg* = *ma*, the masses are canceled out and *a* = *g*. In other words, all objects drop with an acceleration of *g* regardless of their mass (at least in vacuum). While this is true in a homogeneous field, it is more complicated in field geometries that are typical for torsion balances, see below. (3) The measurement required is the angular acceleration of the turntable. The angular acceleration is computed from a time series of angle measurements. Taking the second derivative with respect to time yields the angular acceleration. Similar to the static deflection method, a precise measurement of an angle is needed. Here, however, the experiment can be constructed in such a way that a full circle (2*π*) can be measured. This provides a self-calibration point and can even be used to characterize non-linearities of the angle encoder.

**FIG. 9.**
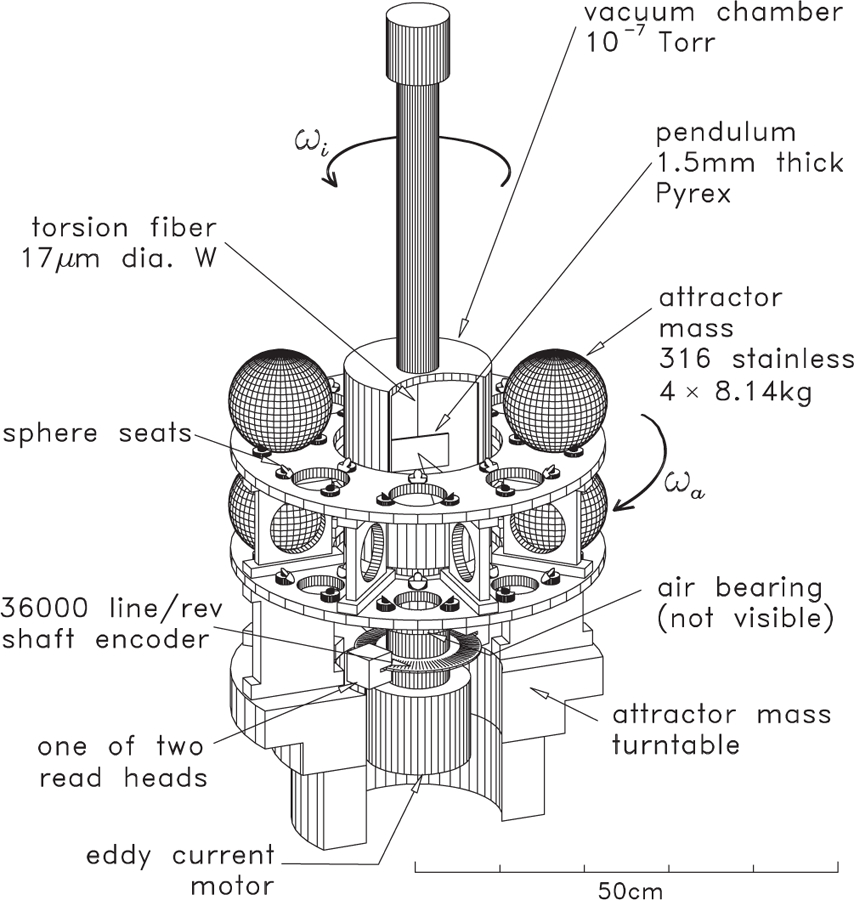
Cut-away drawing of the torsion balance used by Gundlach and Merkowitz to determine *G*. This instrument has measured *G* with the smallest relative standard deviation to date, 13.6 × 10^−6^. Reprinted with permission from J. H. Gundlach and S. M. Merkowitz, Phys. Rev. Lett. **85**, 2869–2872 (2000). Copyright 2000 American Physical Society.^53^

To date, this method has produced the measurement of *G* with the smallest uncertainty. The experiment was carried out by Gundlach and Merkowitz at the University of Washington in 2000.^53^ Figure 9 contains a three-dimensional drawing of the experiment. This experiment incorporates several sophisticated techniques in addition to those mentioned above. For example, the outer masses are also mounted on a turntable whose angular velocity is controlled such that the difference in angular velocities between the inner and outer turntables remains constant. The relevant signal is only at the difference frequency, while most parasitic couplings occur on the rotation frequency of the inner turntable.

A dramatic decrease in uncertainty over previous experiments was achieved through an innovative shape of the pendulum bob. Unlike prior experiments that used dumbbells or cylindrical rods as test masses, the duo at the University of Washington used a thin rectangular plate. This geometry makes the experimental result almost independent of the detailed mass distribution. An elegant way of performing the mass integration for an experiment with a rotation axis is via the multipole formalism.^88,94^ The torque on a pendulum is given by

(27) $$N\left(\phi \right)=-4\pi G\sum_{l=2}^{\infty }\frac{1}{2l+1}\sum_{m=-l}^{l}mq_{lm}Q_{lm},$$

where *ϕ* is the angle between the field mass and test mass assemblies. *q_lm_* denotes the pendulum’s multipole moments and *Q_lm_* denotes the multipole fields of the source mass arrangement.^94^ Well below the resonance frequency of the pendulum, this torque produces an acceleration of the pendulum given by *ϕ* = *N*/*I*. For conventional mass distributions (field masses at a larger radius than test masses), the series in Eq. (27) converges quickly. Thus, the largest term is given by the product of *q*_22_ and *Q*_22_.

For a thin plate with mass *m*, width *b*, and thickness *d*, the inner multipole moment is given by^95,96^

(28) $$q_{22}=\frac{\sqrt{5}m}{16\sqrt{6\pi }}\left(b^{2}-d^{2}\right).$$

The moment of inertia of this plate is given by

(29) $$I=\frac{m}{12}\left(b^{2}+d^{2}\right).$$

Combining Eqs. (27)–(29) yields

(30) $$\underset{d\to 0}{\lim\;}\alpha_{22}\left(\phi \right)=-\sqrt{\frac{24\pi }{5}}GQ_{22}\sin2\phi .$$

For a two-dimensional plate, the angular acceleration is independent of its dimensions and mass. For a pendulum with finite thickness, the expression in the right-hand side of Eq. (30) has to be multiplied by the correction factor

(31) $$\frac{b^{2}-d^{2}}{b^{2}+d^{2}}.$$

The angular acceleration remains independent of the mass of the pendulum bob. Gundlach and Merkowitz chose *b* = 76 mm and *d* = 1.5 mm that minimized higher-order contributions to the series in Eq. (27). With these dimensions, a correction factor of 1–7.8 × 10^−4^ is obtained. Since the correction deviates by only a small amount from one, pendulum’s dimensions do not have to be known precisely. The relative standard deviation contribution to the test mass metrology was 4 × 10^−6^, for a total relative standard uncertainty of 14 × 10^−6^. By way of comparison, the experiment carried out by Luther and Towler^50^ obtained a total relative uncertainty of 64 × 10^−6^. The sum of the contribution of the metrology of the small mass system was 48 × 10^−6^. The knowledge of the mass distribution of the pendulum bob was the dominating component in the uncertainty budget of Luther and Towler. By using a flat plate, Gundlach and Merkowitz managed to reduce this component significantly.

### B. One pendulum or two pendulums

#### 1. From the clock to the gravimeter

Timekeeping is a very important task in science and technology, as well as in commerce. Accurate navigation, for example, is impossible without accurate clocks. The search for stable clocks therefore goes back almost to the dawn of human civilization itself. In 1657, Christian Huygens patented the pendulum, which at the time was considered a very stable clock. He also described the motion of the pendulum. Around 1672, Jean Richer noticed on a trip to French Guiana that the oscillation frequency of a seconds pendulum depends on the geographical latitude. The dependence of the local acceleration on the latitude is a consequence of the Earth’s rotation: The local acceleration is a sum of the centrifugal acceleration and the gravitational acceleration. At the equator, the local acceleration is reduced by the centrifugal acceleration. This effect is exacerbated by the fact that the figure of the Earth is in response to the centrifugal acceleration an oblate spheroid. Hence the polar radius is smaller than the equatorial radius, increasing the gravitational part of the local acceleration towards the pole. A model describing this normal gravity *g*_0_ approximately, the so-called reference ellipsoid, is WGS84,^97^

(32) $$g_{o}=g_{s}\left(\frac{1+a\;\sin^{2}\phi }{\sqrt{1-b\;\sin^{2}\phi }}\right),$$

where

$$g_{s}=9.780\;326\;77\;1\;4\;\text{m}\;{\text{s}}^{-2},\;\;a=0.001\;931\;851\;386\;39,\;\;\;b=0.006\;694\;379\;990\;13,$$

and *ϕ* denotes the latitude. This formula describes the theoretical local acceleration on an equipotential surface at mean sea level. It includes both gravitational and centrifugal potentials. In the 18th century, a debate took place as to whether the radius of the Earth was greater at the equator or at the poles. To resolve this debate, the King of France, Louis XV, sent two groups of scientists to take measurements. One group traveled to Lapland, near the North Pole, and the second group to Ecuador (then called the Territory of Quito by Spain), close to the equator. A prominent member of the second expedition was Bouguer, a French scientist and geodesist who, besides the measurements of arcs of the Earth’s curvature, conducted two experiments to determine the mean density of the Earth.^98^ The first measurement was performed in Quito, which can be thought of as being located on top of a plateau. He measured the period of the pendulum at this location and compared it to the period determined at sea level. The period of a simple pendulum in local gravity *g* is

(33) $$T_{0}=2\pi \sqrt{\frac{l}{g}},$$

with the pendulum length *l*. If there were no mass between both heights, the swing rate difference would be that derived from Newton’s inverse square law, the so-called *free*-*air correction*. The measured gravity, however, was higher. Bouguer explained this difference by assuming the mass between Quito and the sea level was a slab of Earth of equal density. The attraction from this slab can be approximated by

(34) $$\Delta g_{\text{B}}=2\pi G\rho H,$$

where *ρ* is the density of the Earth and *H* is the height of the slab. The correction described by Eq. (34) is still applied in geophysics today and is called the *Bouguer correction* or *Bouguer anomaly*. From this measurement, Bouguer could determine the mean density of the Earth. If the mean density of the slab is known (for example, from measurement), then this density can be compared to the mean density of the Earth—similar to what Cavendish did, when he compared the mean density of the Earth to that of the field mass. Bouguer estimated the mean density of the Earth as being more than 4 times that of the slab. The standard density of rock in geophysics, as used in the Bouguer correction, is 2.67 g cm^−3^. The mean density of the Earth, however, as derived from the current *G* value, is about 5.5 g cm^−3^. Bouguer’s estimation was obviously too high.^11^

**FIG. 10.**
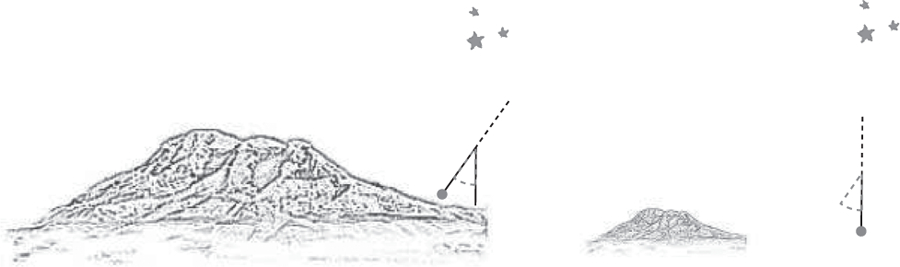
Principle of the pendulum measurement performed by Bouguer. He measured the attraction of Mount Chimborazo to the pendulum bob: first close to the mountain (left) and then far from the mountain (right). The plumb line is measured with respect to the fixed stars.

In another experiment, he measured the horizontal attraction of Mount Chimborazo, a mountain more than 6000 m high. The principle of the experiment is depicted in Fig. 10. Here as well, two measurements were necessary. In the first measurement, Bouguer placed the pendulum at the foot of the mountain and measured the direction of the plumb line with respect to the stars. In the second measurement, he placed the pendulum farther away from the mountain, but at the same latitude [normal gravity changes with latitude due to the shape of the Earth; see Eq. (32)], and again measured the direction of the plumb line with respect to the stars. The angular difference was a measure of the pull of the mountain onto the pendulum bob by comparison with a model based on Newton’s law of gravitation.^11^ Neither of the two experiments gave even an approximate mean density of the Earth, but this is extremely difficult, since local density inhomogeneities can lead to large errors. Several similar experiments were later performed by other scientists (e.g., Maskelyne’s experiment at Mount Schehallien, a mountain in Perthshire, Scotland, in 1774^99^). High accuracies were not expected, as the “field mass” (i.e., the Earth) has a very irregular density distribution. Well-defined field masses are required in addition to good environmental conditions.

#### 2. A modern experiment measuring the time of swing of a simple pendulum

A modern version of measuring *G* by observing the period of two simple pendulums in vacuum is currently being built at the Politecnico di Torino, Italy by De Marchi.^100^ Two 1m long pendulums spaced 0.1 m apart measure the change in local acceleration introduced by a field mass arrangement. The field masses are moved such that they slow the period of either one pendulum or the other. The relative frequency difference is modulated with the field mass position. This differential measurement rejects several common mode effects, e.g., changes in the gravitational environment identical to both pendulums due to tides or moving masses in the laboratory. The field mass induced frequency change is of order 10^−7^ of the resonance frequency. In order to resolve this frequency change, a very high quality factor is required. De Marchi is aiming to reach a quality factor in the order of 10^8^. Such a high quality factor would allow a measurement with a relative uncertainty of 10^−5^.

#### 3. Modern experiments measuring the static deflection with two simple pendulums

**FIG. 11.**
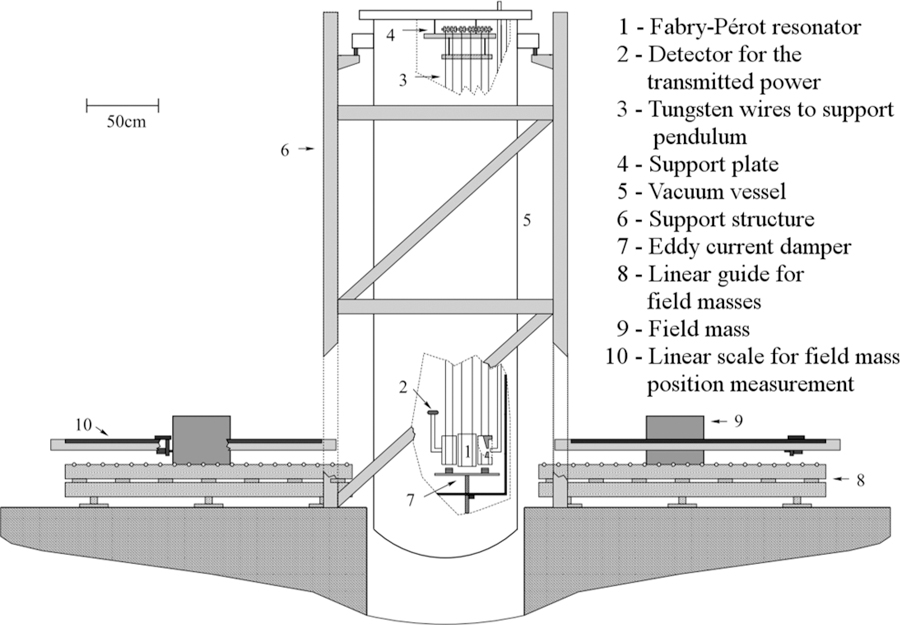
A laboratory version of Bouguer’s pendulum experiment. Two field masses attract the bobs of two pendulums, which form a microwave cavity. Reprinted with permission from U. Kleinevoß, “Bestimmung der Newtonschen Gravitationskonstanten *G*,” WUB-DISS 2002–2, Ph.D. thesis (University of Wuppertal, 2002).^102^

A laboratory version of this Mount Chimborazo experiment was conducted in 2002 by Kleinevoß *et al.*^101,102^ Their setup, which is depicted in Fig. 11, consisted of two pendulums of length l≈2.6 m. The bobs of these pendulums formed a microwave resonator. Two symmetrically arranged field masses, each of which was made of brass and had a mass of about 576 kg, were placed alternating at two distances from the bobs. The microwave frequency measured when the field masses were far (≈2.1 m) from the pendulums was taken as a reference, *f*_ref_. When the field masses were placed closer (≈0.6 m) to the pendulums, the frequency, *f*_meas_, of the resonator changed due to the attraction of the field masses, which made the two mirrors separate more, thus leading to a different cavity length. The frequency difference ∆*f* = *f*_meas_ − *f*_ref_ was then used to determine *G* with

(35) $$\Delta f=\left(\frac{df}{db}\right)G\frac{M}{\omega_{0}^{2}}[\left(\frac{1}{r_{\text{meas}}^{2}}-\frac{1}{{\left(r_{\text{meas}}\right)}^{2}}\right)K_{\text{r}}\;\;\;\;\;\;\;\;\;-\left(\frac{1}{r_{\text{ref}}^{2}-\frac{1}{{\left(r_{\text{ref}}+b\right)}^{2}}}\right)K_{\text{ref}}],$$

where *M* denotes the masses of the field masses, *ω*_0_ denotes the fundamental frequency of the pendulums oscillations, *b* denotes the cavity length, and *K*_r_ and *K*_ref_ are correction factors for the field mass distributions. The relative combined standard uncertainty they reached was 147 ppm.^102^

In 2010, Parks and Faller published the results of a similar experiment,^103,104^ which had actually been conducted in 2004. Two main differences to the Wuppertal experiment are noteworthy. The first difference was the use of a laser Fabry-Pérot interferometer (cavity), rather than a microwave cavity. Reducing the wavelength of the electromagnetic waves from centimeters to several hundreds of nanometers improved the resolution of the distance sensing. The second difference was a simultaneous measurement of a second cavity, which was attached to the support of the pendulums. This was done in order to compensate thermal drifts in the setup. Their pendulums had a length of 72 cm and four identical source masses of 120 kg each were used. The group waited six years before publishing the results, as the value of *G* they found was about 14 standard deviations higher than the CODATA-2008 value. Before submitting the publication, every detail of the experiment was carefully checked. The final result has a relative standard uncertainty of 21 × 10^−6^.

### C. Conventional balances

#### 1. Historical beam balance measurements

A conventional beam balance compares the clockwise and counterclockwise torques generated by masses on two balance pans on either side of the fulcrum. The beam tilts until the net torque about the central pivot is zero. Traditionally, the center of mass of the beam is below the pivot point and, as the beam pivots, a counteracting torque is generated. The tilt of the beam is a measure of the initial torque difference. Assuming that the arms are of equal length, the beam balance is a device to compare forces. The assumption of equal arm lengths is not necessary if a weighing scheme such as substitution or transposition is implemented.

The weight of a mass is given by the product of the mass value and the local acceleration of gravity, *mg*. In most cases, balances are used to measure *m*, since *g* is assumed to be constant. In measurements of *G* with beam balances, *m* is constant and changes in *g* introduced by modulating a source mass are measured. The readings of beam balances are usually given in grams and must be converted into the units of force by multiplying them by the local acceleration.

**FIG. 12.**
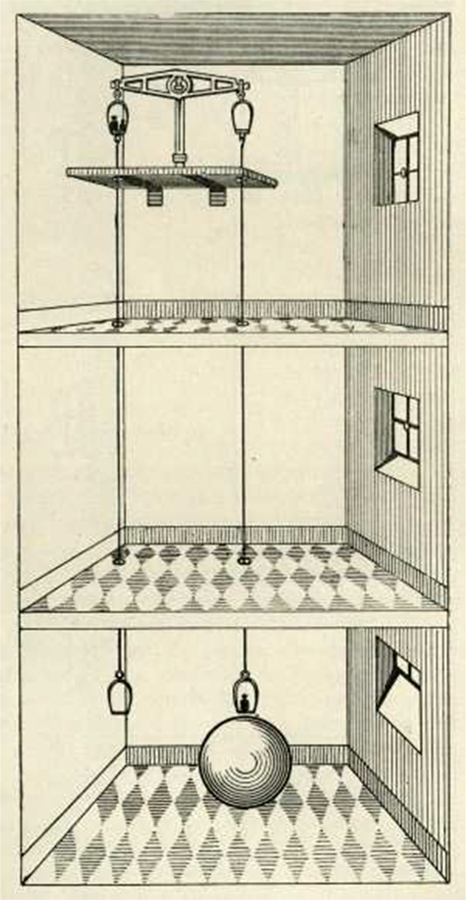
Double-balance of the German physicist von Jolly. From Graetz, *Die Physik*. Copyright 1917 Max Planck Institute for the History of Science. Reprinted with permission from Max Planck Institute for the History of Science.^108^

The beam of a balance with two identical masses on the balance pans is in equilibrium. Changing the local gravity at the position of one mass causes an excursion of the beam proportional to the weight change. The weight change can either be read off at the balance’s pointer or small calibrated masses can be added to restore equilibrium. Local gravity can be changed by placing large field masses below or above the pans. Von Jolly conducted such a measurement in Munich, Germany, in 1878 and 1881, almost a century after Cavendish.^17,105,106^ He built a special beam balance—a so-called “double-balance”— with four mass pans (see Fig. 12) that was able to resolve weight differences smaller than 1 *μ*g.^107^ Von Jolly was able to measure the Earth’s gravity gradient, as the gravity decreases by about 300 *μ*Gal m^−1^ (in gravimetry, the non-SI unit “Gal” is commonly used: 1 Gal = 1 cm s^−2^). Raising a 1 kg mass by 1 m decreases its weight by about 3 *μ*N (which would correspond to a mass of 300 *μ*g). In von Jolly’s setup, the upper and lower mass were separated by about 21 m, leading to a relative weight change of 6 × 10^−6^. Von Jolly used this experiment to verify Newton’s inverse square law (*F* ∝ *r*^−2^). To do so, four identical glass flasks were made, two of which were filled with approximately 5 kg of mercury and two of which were air-filled. Then, all four flasks were sealed. The purpose of the air filled flasks was to suppress effects related to changes in the air buoyancy due to changing air pressure. For the measurement, von Jolly first placed both mercury-filled flasks on the upper pans and the air-filled flasks on the lower pans. Later, he switched the two flasks on one arm of the beam. As he expected, he was able to measure an increase in weight when the mass was moved from the upper to the lower pan. A difference was observed with respect to theoretical calculations; however, this difference was attributed to mass inhomogeneities in the Earth. For his *G* measurement, he placed a field mass—a lead sphere with a diameter of approximately 1 m and a mass of 5775 kg— below one of the lower pans (see Fig. 12). Then, he applied the same measurement strategy as before.^12^ With a distance between the centers of mass of the field mass and the test mass of *a* = 0.57 m, he measured an increase in weight of 5.8 *μ*N, which corresponds to a mass of 589 *μ*g. This difference corresponds to 10^−7^ times the mass of the test mass. Von Jolly was able to measure this difference with a relative uncertainty of 1.2 %, a highly significant achievement for the time. He obtained *G* = 6.465 × 10^−11^ m^3^ kg^−1^ s^−2^. To be historically accurate, von Jolly did not directly measure *G* but instead the mean density of the Earth, similar to Cavendish. He compared the density of the lead, *ρ*_Pb_, with that of the Earth, *ρ*_Earth_,

(36) $$\rho_{\text{Earth}}=\rho_{\text{Pb}}\left(\frac{m}{\Delta m}\right)\left(\frac{r}{R}\right)\left(\frac{r^{2}}{a^{2}}\right),$$

where *R* denotes the radius of the Earth.

Hermann von Helmholtz later encouraged König and Richarz, who were later joined by Krigar-Menzel,^29,109^ to repeat the measurement with a modified setup in Berlin, where they used the incredible amount of approximately 9 m^3^ of lead. The mass of this field mass was about 100 metric tons of weight. The lead was made available from a nearby cannon foundry. The field mass was a large rectangular block.^17^ The British physicist Poynting measured *G* with a beam balance in 1878.^110^ Like von Jolly, he used a spherical lead mass as a field mass, although with a smaller mass (170 kg). None of these experimenters reached the uncertainty that von Jolly reached.

#### 2. Modern beam balance measurement

The next time a beam balance was used to measure *G* was almost a century later although, beam balance experiments to measure other aspects of gravity took place in the early 1900s. In 1983, Speake wrote a Ph.D. thesis on the beam balance method to determine the Newtonian gravitational constant.^111,112^ More recently, experiments were conducted by a group at the University of Zurich led by Kündig (see Refs. 113 and 114). The aim of the first measurement was to search for a fifth force with a range of about 100 m. The experiment, which was conducted at the Gigerwald storage lake, found the value for *G* for interaction ranges of 88 m and 112 m^115,116^ to be consistent with the values obtained in laboratory experiments (with interaction ranges typically on the order of 10 cm). The largest uncertainty in the measurement of *G* was the mass integration. The shore of the lake was composed of scree and it was difficult to know where the water ended (i.e., the source mass was ill defined).

The obvious solution was to “bring the lake to the laboratory.” Since water has a low density, a huge volume of water would have been necessary to create a sizable signal. For this reason, Kündig *et al.* resorted to a material that von Jolly had used: mercury. However, this time, mercury was used as a field mass. Mercury has a density of 13.54 g cm^−3^, which allows it to be used very effectively as a field mass. Effective, in this context, means that a small volume of the field mass can create a large signal.

The second desirable property of mercury is that it is liquid at room temperature. Liquids have a higher homogeneity in density than solids. The density in solid metals can vary, depending on the details of the casting process, relatively up to 10^−4^. The mass distribution of a liquid field mass has (almost) perfect homogeneity. Possible deviations from perfect homogeneity include a linear density gradient due to the isobaric pressure gradient and the compressibility of the liquid. The major disadvantage of a liquid field mass is that a tank, which must be taken into account for the mass integration, is needed to contain the liquid. A tank is usually a very complicated structure consisting of several parts that are joined by bolts and use O-rings in grooves to seal the joints. The mass, shape, and location of these elements must be measured, and their effect on the gravitational signal must be calculated, increasing the complexity of the mass integration compared to an experiment with a solid source mass.

**FIG. 13.**
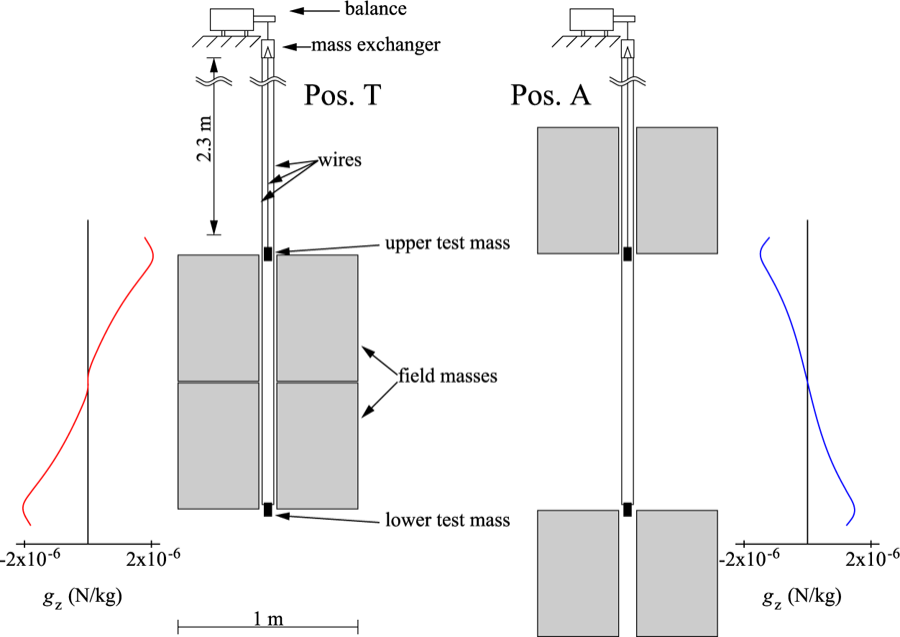
The principle of the experiment conducted at the University of Zurich. The two gray cylinders (field masses) can be either together (T) or apart (A). Either one of the two test masses is connected to the mass comparator to measure its weight given by *m*(*g* + *g*_*z*_ ), where *g* is the local acceleration of gravity at the test mass position and *g*_*z*_ is the additional vertical field produced by the source masses. On either side of the drawing, *g*_*z*_ is shown. Reprinted with permission from S. Schlamminger *et al.*, Philos. Trans. R. Soc., A **372**(2026), 20140027 (2014). Copyright 2014 The Author(s) Published by the Royal Society.^117^

In the Zurich experiment, a total of 1 m^3^ of mercury was filled into two identical tanks shaped like hollow cylinders (see Fig. 13). It is possible to move the tanks into either of two positions, labeled “together” and “apart.” A vacuum tube passed through the inner bore of the tanks. Inside the vacuum tube are two test masses suspended by tungsten wires. Vertically, the test masses are separated by 1.4 m, approximately double the height of one tank. Thus, when the field masses are together, each test mass is located at the end of a field mass cylinder. The gravitational field generated by the cylinder has an extremum at this position (see Sec. V). The “apart” position is designed in such a way that each test mass is again at an extremum of the field. In the together (T) and apart (A) states, the force difference between the upper (*m*_u_) and lower (*m*_l_) masses is measured as follows:

(37) $$\Delta F_{\text{T}}=m_{\text{u}}g\left(z_{\text{u}}\right)+F_{z}\left(\text{T},\text{u}\right)\;\;\;\;\;\;\;\;\;\;\;-m_{\text{l}}g\left(z_{\text{l}}\right)-F_{z}\left(\text{T},\text{l}\right),$$

and

(38) $$\Delta F_{\text{A}}=m_{\text{u}}g\left(z_{\text{u}}\right)+F_{z}\left(\text{A},\text{u}\right)\;\;\;\;\;\;\;\;\;\;\;-m_{\text{l}}g\left(z_{\text{l}}\right)-F_{z}\left(\text{A},\text{l}\right),$$

where *F_z_*(A/T, u/l) denotes the vertical force on the upper/lower test mass in the field mass state (apart/together). *g*(*z*_u/l_) denotes the local acceleration of gravity at the position of the upper/lower test mass.

Subtracting the differences from each other eliminates the weights *m*_u_*g*(*z*_u_) and *m*_l_*g*(*z*_l_) and yields the following:

(39) $$\Delta F_{\text{T}}-\Delta F_{\text{A}}=F_{z}\left(\text{T},\text{u}\right)-F_{z}\left(\text{A},\text{u}\right)-F_{z}\left(\text{T},\text{l}\right)+F_{z}\left(\text{A},\text{l}\right)\;\;\;\;\;\;\;\;\;\;\;\;\;\;\;\;\;\;=kG,$$

where *k* denotes a factor containing masses and distances between masses. This second difference, also referred to as the signal, was approximately 8 *μ*N.

The vertical forces on the test masses are measured by a very sensitive mass comparator. A mass exchanger hanging below the mass comparator can connect either mass to the mass comparator. The reading of the mass comparator is in units of mass (kilogram) rather than force (Newton). To obtain the gravitational force, the reading has to be multiplied by the measured value of *g*.

In contrast to torsion balance experiments, the traceability of the force measurements in beam balance experiments is straightforward: A traceable calibration force can be generated by adding a mass standard with a calibrated mass to the balance pan. The second measurement required is that of *g*, which is not usually a problem at relative uncertainties of 10^−6^.

The relative uncertainty obtained by the Kündig experiment was 18.3 × 10^−6^. The uncertainty was limited by the statistical uncertainty (i.e., the noise in the weighing and sorption effects on the test masses that are correlated to the motion of the field masses).

The Zurich experiment was dismantled at the end of 2002, and mercury was sent back to the mine it was leased from.

### D. Free-fall absolute gravimeters and gradiometers

#### 1. Principle of free-fall gravimeter

**FIG. 14.**
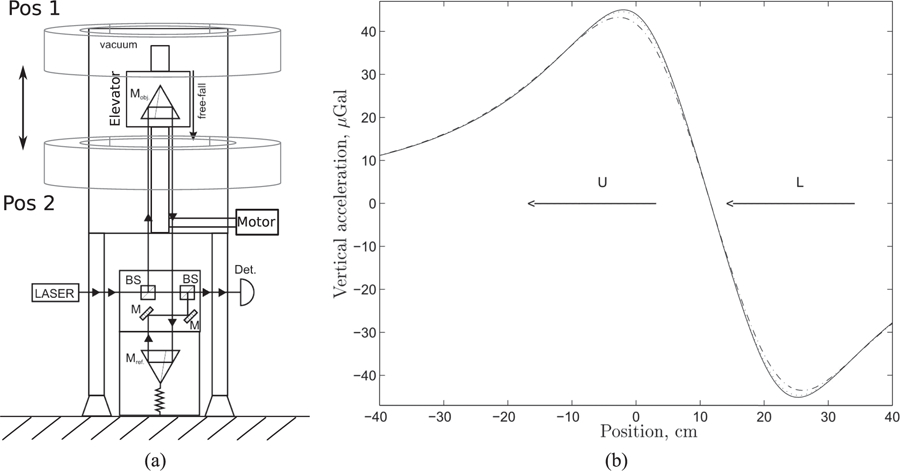
(a) In a free-fall absolute gravimeter, a test mass contains a retroreflector *M*_*obj*_, which is part of a Mach-Zehnder laser interferometer. The test mass is released in vacuum and its free-fall path is traced with respect to an inertially isolated reference retroreflector (M_*ref*_ ). BS denotes beam splitters; M denotes mirrors. The interference signal registered with the detector *Det* contains the information about the acceleration due to gravity. For repeated measurements, the test mass is lifted up with an elevator. The alternating positions of a field mass are sketched by the two rings. (b) The graph shows the qualitative field strength of the ring-shaped field mass. Two extrema appear. The trajectory of the test mass is adjusted to precisely cover the range of the extrema in order to minimize positioning errors. When the field mass is in the lower (L/Pos 2) position, the measured gravity is higher than the local gravity. When the field mass is positioned above (U/Pos 1) the test mass, the measured gravity is lower than the local Earth’s gravity. The theoretical effective gravity from the source mass is obtained by integrating over the field strength covered by the trajectory. Reprinted with permission from J. P. Schwarz *et al.*, Science **282**, 2230–2234 (1998). Copyright 1998 AAAS.^118^

An absolute gravimeter is an instrument used to measure the local acceleration due to gravity, *g*, also commonly called “Little *g*.” A diagram explaining the principle of a classical free-fall absolute gravimeter is shown in Fig. 14(a).

A test mass to which a retroreflector is attached is released in a vacuum chamber. The trajectory of the mass in free fall is traced by means of a laser interferometer. In order to conduct repeated measurements, the test mass sits in an elevator. This elevator is accelerated with more than *g* downwards. As a result, the test mass hovers inside the elevator and falls freely without being in contact with the elevator until the elevator decelerates in order to catch the test mass again gently. The accelerated motion of the test mass produces a chirped fringe signal, which is detected with a photo diode. From the fringe signal, the trajectory of the free fall can be recovered, as one fringe crossing corresponds to a travel distance of the test mass of half the laser wavelength. By timing the fringe signal values, the time/distance (*t_i_*, *z_i_*) pairs are obtained and a least-squares fit of a linear model provides the acceleration, *g*_0_,

(40) $$z_{i}=z_{0}+v_{0}\left(t_{i}+\frac{1}{6}\gamma t_{i}^{3}\right)+\frac{1}{2}g_{0}\left(t_{i}^{2}+\frac{1}{12}\gamma t_{i}^{4}\right).$$

The parameters *z*_0_ and *v*_0_ denote the initial position and velocity at the start of the trajectory (i.e., for *t* = 0). There are two ways to include the Earth’s gravity gradient, *γ*: The first way is to include the gradient (if it is known), as in Eq. (40) in the fit model. Then, the calculated acceleration refers to the start position of the trajectory. The second way is to skip the gradient in the fit model (i.e., by setting *γ* = 0). In this case, the calculated acceleration refers to a position between the start and the end of the trajectory, a function which depends on the initial velocity, *υ*_0_, of the test mass, the approximated local gravity, *g*_0_, and the total free-fall time, *T* (see, e.g., Ref. 119). This position is then called the reference or reported height.^120^ Resolutions on the order of 1 part in 10^9^ and better are possible. A short overview of relative and absolute gravimeters can be found in Ref. 121.

From a didactic standpoint, free-fall experiments are ideally suited to measure *G*. After all, Newton was allegedly inspired to derive the law of gravitation after observing the free fall of an apple from a tree. However, free-fall experiments also have a practical advantage over the experiments discussed above. Free-fall experiments do not require a suspension for the test mass(es). Perturbing material properties, which are often not well understood or whose models are only valid to a certain degree, do not apply here. A disadvantage, on the other hand, is the short measurement time of such experiments in an Earth-bound laboratory. In a satellite encircling the Earth, this situation would be different, as mentioned above.

#### 2. G measurement with a free-fall gravimeter

**FIG. 15.**
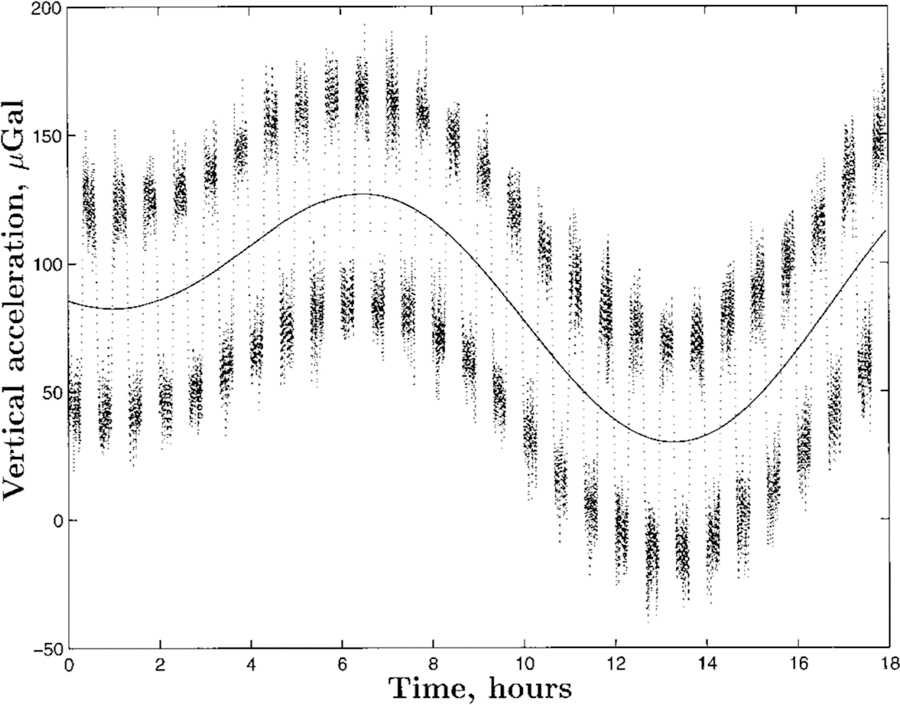
The differential signal of the *G* experiment with a gravimeter. The gravity was measured for two different field mass positions. The time variation in the gravity signal arises mainly from the Earth tides (solid middle line). Reprinted with permission from J. P. Schwarz, Science **282**, 2230–2234 (1998). Copyright 2015 AAAS.^118^

*G* can be measured with a gravimeter by placing a well-defined field mass close to the gravimeter’s test mass. Because the field mass perturbs the local acceleration due to gravity, the gravity measured is *g*_meas_ = *g*_0_ + *g*_FM_ (i.e., the local Earth’s gravity, *g*_0_, plus a perturbing term arising from the field mass, *g*_FM_). By applying Newton’s law of gravitation, this perturbation can be modeled, and the acceleration measured can be taken to determine the magnitude of *G*. This experiment was conducted in 1998 by a group led by Faller at Boulder, USA, using a commercial free-fall absolute gravimeter FG5.^118^ Twelve tungsten alloy cylinders (in two layers) were placed to form a ring. The gravity field of a ring mass shows two extrema where the integrated signal over the test mass trajectory reaches its maximum, see Fig. 14(b). Another advantage of the ring shape is that the field strength variation at these extrema is minimal; as a result, a minimum uncertainty due to positioning errors is given. The relative standard uncertainty obtained was on the order of 1.4 × 10^−3^. This is only approximately five times better than what Cavendish obtained, but the important point is that it represents a completely different measurement approach. It does not need to compensate for Earth’s gravity by means of (for example) a torsion wire, an error source that was underestimated for many years.^36^ Although there is no drift due to material properties, many other factors are part of this relatively high uncertainty. First, there is a poor signal-to-noise ratio. Although gravimeters can resolve gravity to better than one part in 10^9^, the signal, when compared to *g*, is only about 1 part in 10^7^. This requires a long integration time. In order to compensate for systematic effects, the experiment was repeated with two different field mass positions. In the first mode, the field mass was placed below the test mass; as a result, the gravity measured was higher than *g*_0_. In the second mode, the field mass was placed above the test mass, thus taking advantage of both extrema of the ring-shaped field strength. This second position reduced the effective gravity acting on the test mass. Figure 15 shows some measurement data of the experiment. The time variations in the data are mainly from the influences of the Moon and the Sun (tides), as well as due to environmental effects (e.g., temperature, air pressure, and hydrological effects). The pure tidal variations are already three times the magnitude of the field mass signal. Moving the 500 kg field mass produces additional scatter on the data.

#### 3. Cold atom gravimeters and gradiometers

Dropping a macrosopic retroreflector is not the only way to realize a free-fall gravimeter. In 1991, Kasevich and Chu^122,123^ were able to measure *g* by dropping cooled atoms (i.e., by using atom interferometry^124^). Here, a cloud of atoms are cooled and all atoms are prepared to the same quantum state, described by their phase *ϕ*. Then, the atoms are released by briefly turning off the trap. Three laser pulses with a pulse separation *T* follow. The first pulse splits the cloud into two by increasing the momentum of some of the atoms. This laser pulse is called a *π*/2 pulse. The second pulse acts on both clouds in such a way that their momenta are interchanged (the so-called *π*-pulse). Finally, the third pulse, which is a second *π*/2 pulse, recombines both clouds. As both clouds interact at different heights with the laser, where the clouds have different velocities and the laser has a different phase, the states of the atoms are different, depending on the acceleration.

The final recombined cloud is then interrogated by another laser in order to obtain the information on the atom quantum states. The probability *P* = (1 + cos(*ϕ*))/2 of how many atoms are still in their initial state is then a measure of *g*. Here, *ϕ* represents the total accumulated phase difference. Although the information about the acceleration is extracted from the quantum states of the atoms, the principle has some points in common with the macroscopic setup. It should be noted that atom gravimeters essentially correspond to a measurement of three positions of the atom clouds—or to a measurement of two velocities. During the pioneering years of conventional free-fall gravimeters, only three points were measured, whereas today, data have been collected on thousands of positions.^121^ These developments took place before lasers were available. Moreover, the measurement sequence *π*/2 − *π* − *π*/2 corresponds to a Mach-Zehnder laser interferometer type, the interferometer setup that is commonly used in conventional gravimeters. From these commonalities, it follows that perturbations enter both systems in the same way, as explained in Ref. 120. This can be easily seen by comparing of the measurement functions, which can be written as

(41) $$g_{\text{meas}}=\frac{1}{k}\frac{\phi_{1}-2\phi_{2}+\phi_{3}}{T^{2}},$$

where *T* denotes the total free-fall time. For a classical gravimeter, the factor *k* = 4*π*/*λ*, with *λ* being the wavelength of the laser (usually 633 nm). For an atom gravimeter, *k* = *k*_eff_ (the effective Raman wavenumber). In a conventional gravimeter, *ϕ_i_* denotes the time-dependent phase difference between both interferometer beams; in an atom gravimeter, it denotes the local Raman phases at the times when the *π*/2 and *π* pulses are applied. Also of note is the fact that atom gravimeters can be constructed in two ways: using a simple drop of atom clouds and using a fountain-like setup (i.e., the launch-and-drop method), which is actually the most common method for atom gravimeters. The same is true for conventional gravimeters although, here, the simple free fall is preferred.

Atom gravimeters have already been used in several *G* measurements in different configurations.^125^ However— possibly as a consequence of the knowledge gained from classical gravimeters—a simple atom gravimeter configuration has never been used for *G* measurements but a combination of two gravimeters, defining a gradiometer. A gradiometer measures spatial gravity differences; for example, if the setup depicted in Fig. 14(a) is imagined with the reference retroreflector M*_ref_* also in free fall (as suggested in Ref. 118), both retroreflectors fall at the same time with a spatial separation *d* along the plumb line. Since the lower retroreflector is closer to Earth’s center of gravity, its acceleration due to gravity will be larger than the upper retroreflector. If this differential acceleration ∆*g* is measured, then the Earth’s gravity gradient *γ* = ∆*g*/*d* can be calculated together with the known separation *d*. Since both masses fall simultaneously, environmental changes or tidal signals are common mode effects for both masses and are thus canceled out. Such a gradiometer would give an improved signal-to-noise ratio, as the background signal is no longer *g* but only its gradient *γ*, which is on the order of 3 × 10^−7^ m^−1^. At this point, *G* is measured by perturbing the local gravity gradient by adding a well-defined field mass, as in the gravimeter experiment. However, the Earth’s gravity gradient can only be measured to a few parts in 10^−2^, which limits the achievable accuracy of the experiment. In order to circumvent this problem, an additional differential measurement can be conducted. By repeatedly positioning the field mass at different positions, the differential signal can be considered. An experiment of this type was carried out by Fixler *et al.*,^126^ who achieved a relative uncertainty of 5 × 10^−3^, and more recently by Rosi *et al.*,^127^ who gave a relative uncertainty estimation of 1.5 × 10^−4^.

#### 4. Differential gravity gradiometer

**FIG. 16.**
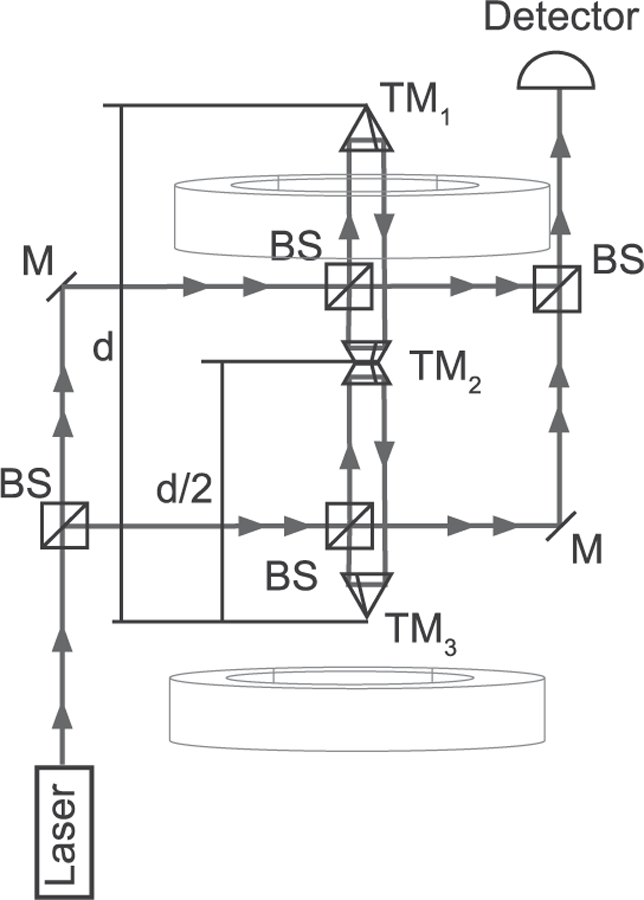
Differential gradiometer principle. Test masses TM_1_, TM_2_, and TM_3_ are in simultaneous free fall. The ring-shaped field masses perturb the local gravity field. Due to the differential character of the setup, only the gravity of the field masses is measured, not the Earth’s gravity or its gradient (to first order) (M—mirror, BS—beam splitter, d—distance between upper and lower test mass).

The gradiometer setup still involves the problem of an unknown gravity gradient; the differential measurement requires a re-positioning of the field mass, which leads to positioning uncertainties. One way of avoiding this problem was proposed in 2014 by Rothleitner and Francis^128^ and combines two gradiometers in one, as depicted in Fig. 16. The second (middle) test mass (TM2) is part of both gradiometers. When all three test masses fall simultaneously and the distance between TM1 and TM2 is the same as that between TM2 and TM3, both gradiometers measure the same (Earth’s gravity) gradient. When taking the differential signal of both gradiometers into consideration, this gradient is canceled to first order and the resulting instrument can be considered a “null instrument.” Such an instrument is highly sensitive to local gravity variations and not influenced by environmental changes since most influences are common mode effects on both gradiometers. This instrument is perfectly suited for measuring local gravity variations and thus for determining *G*. In principle, not even the positions of the field mass have to be varied, as only the influence of the field mass is measured, in accordance with the nature of the instrument. Most of the effects that have a large uncertainty contribution in gravimeter measurements are the common mode here and are canceled out. As a consequence, this setup requires lower stabilities in the laser system and other components, making it less expensive than an absolute gravimeter. Only a few effects are not common mode effects such as the rotation of the test masses.^129^ The test mass contains a retroreflector attached to a housing. If the test mass falls freely, there will always be a minute induced rotational velocity. This rotation will be around the center of mass (COM) of the test mass. If the optical center (OC) differs from the COM, then a parasitic acceleration will be measured (the centrifugal acceleration of the test mass). Thus, the test masses must be well balanced in order for the COM to coincide with the OC.^130^

An experiment with a conventional free-fall gradiometer, as suggested in Ref. 128, has still not been realized. However, a first attempt by means of an atom gravimeter configuration has been carried out by Rosi *et al.* in 2015.^131^

## V. THE MASSES

Besides length and time, the third dimension that appears in the unit of the gravitational constant is mass. The choice of the material and the shape of the test and field masses are not trivial and depend on many factors. Although mass can be measured very accurately by means of comparators, in “Big *G*” measurements, the determination of the mass alone is not sufficient. This is due to the fact that the field mass is usually very close to the test mass for a *G* measurement. As a consequence, we cannot treat the masses as point masses and have to integrate over both mass density distributions from the test and field masses. Therefore, knowledge of the density distribution within the masses is also necessary. Furthermore, highly accurate dimensional measurements are necessary, as well as precise positioning of the test and field masses. Table III shows some examples of field masses that have been used for *G* measurements. The table also shows the very wide range of weights that have been used for laboratory measurements over the centuries. From Eq. (1), we infer that in order to increase the signal, the masses must be as large as possible. At the same time, the distance between the masses should be as small as possible for the signal decays with the inverse square of the distance. This brings us to the conclusion that the density of the material should be maximized. This is why most of the experiments use highly dense material. Elements such as lead, tungsten, and uranium have been used for their high densities. Some of these materials, however, are either hard to process (tungsten) or are relatively soft (lead) and allow the surface to be easily damaged. Because density distribution is also of critical importance, either the mass should be homogeneous or (as a minimum) the density distribution should be easily measurable. Due to the high density of the materials, X-ray imaging methods cannot be used. Therefore, one of the masses used in the experiment is usually examined by means of destructive testing. A better solution is the use of a liquid such as mercury^114^ or water, as they have an almost perfectly homogeneous density distribution. However, because mercury is poisonous, it is generally avoided, while the low density of water means that it can only be used in enormous quantities such as a lake^116,134^ or the sea.^135^

Another important consideration is the shape of the mass. Table III lists four types of shapes: a sphere, a cylinder, a ring, and a block. Spheres have several advantages over other geometrical shapes of the field mass:

1. The sphere is the shape for which the gravitational field can be calculated most easily. The calculation of the gravitational field of other shapes is more involved and sometimes cannot be solved in closed mathematical form.
2. The sphere has the highest symmetry. It can be oriented in many different positions in order to average out density non-uniformity.
3. It is easier to fabricate a large sphere with low form deviations than, for example, a cylinder. Currently, the best spheres in the world are those fabricated for the Avogadro project. These spheres are made from a crystal of almost pure ^28^Si silicon. Their form deviations are on the order of 40 nm (and below) for diameters of 93.7 mm,^136^ and their surface roughnesses are less than 0.2 nm^137^ (i.e., the relative form deviations are about 4.3 × 10^−7^). The tungsten-sphere field masses with nominal diameters of 101.6 mm that were used by Beams^138^ had form deviations of less than 75 nm (relative form deviation of 7.4 × 10^−7^).^133^ For comparison, industrial ball bearings with nominal diameters of up to 100 mm have tolerances in diameter of up to 1 *μ*m (grade 40) (i.e., one part in 10^5^ relative form deviation), whereas smaller ball bearings with nominal diameters of up to 12.7 mm have relative form tolerances of 6.3 × 10^−6^ (grade 3).^139^ In order to estimate the measurement error due to these form deviations, we assume an ellipsoidal shape of the field mass. The relative error can then be calculated as (see Ref. 43)

(42) $$\frac{\Delta a_{\text{form}}}{a_{\text{form}}}=\frac{3\cdot \left(2c^{2}-a^{2}-b^{2}\right)}{10\cdot R^{2}},$$

where *a*, *b*, and *c* denote the radii of the three rotational axis of the ellipsoid and *R* denotes the distance between the test mass and the field mass. If the separation *R* = 100 mm, *a* = *b* = 50 mm, and *c* = *a* − 0.5 *μ*m (grade 40), then this relative error amounts to about 3 × 10^−6^ (i.e., sufficient for a measurement on the order 1 × 10^−5^).

Although it is sometimes believed that a cylinder is easier to manufacture than a sphere, this does not hold if small tolerances have to be met, as the form deviations of cylinders are usually higher than those for spheres. As an example, Tino *et al.* (Florence, Italy) used cylindrical field masses made of 95.3 % W, 3.2 % Ni, and 1.5 % Cu. The relative form deviation, which was measured with a coordinate measuring machine, was on the order of 1 × 10^−5^.^127,140^ While the tolerances for a sphere can be specified with one parameter (radius), additional parameters are necessary to specify the tolerances for a cylinder: cylindricity, parallelity (of end planes), flatness (of end planes), and angles. Hence, it is more difficult to manufacture and to quantify the form deviations on a cylinder. It is challenging to keep the aforementioned tolerances below 1 *μ*m for cylinders.^141^

**FIG. 17.**
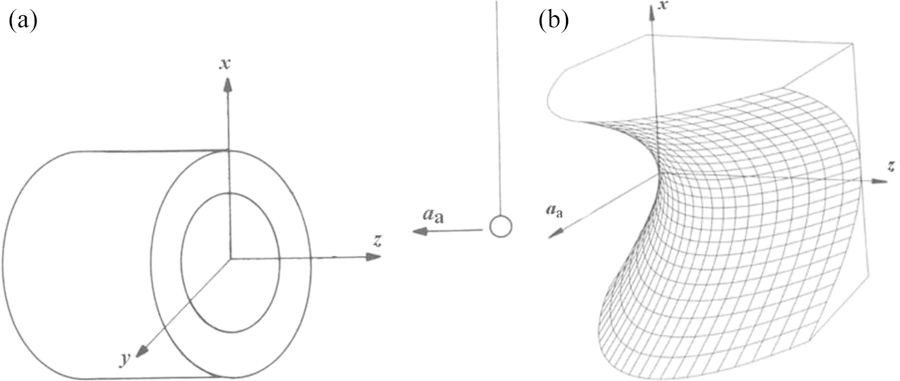
(a) This diagram shows the gravitational field strength *a*_a_ of the hollow cylinder pictured in (b). (b) The gravitational field of the cylinder in (a): It has a saddle point on the axis of symmetry and near the end of the cylinder. From Y. T. Chen and A. Cook, *Gravitational Experiments in the Laboratory*. Copyright 2005 Cambridge University Press. Reprinted with permission from Cambridge University Press.^43^

A considerable advantage of using a hollow cylinder versus a sphere as a field mass is a larger tolerance for the test mass position, as Faller and Koldewyn^142^ pointed out in 1983. At a certain distance above the cylinder along the symmetry axis, the gravitational field of a hollow cylinder has a maximum. At the maximum, the derivative of the gravitational signal with respect to the axial position is zero; thus, the signal to first order is independent of the precise position of the test mass. Chen and Cook^143^ made a detailed calculation and showed that there is actually a saddle point (i.e., a minimum and a maximum along the radial and axial directions, respectively). Hence, the signal is also independent to first order of the radial position. Figure 17 depicts a sketch of this saddle point. Kündig *et al.* also took advantage of this property, as shown in the graphs on the left and the right of Fig. 13.

Such a field mass can also be constructed by arranging a number of cylinders to form a ring, as done in the free-fall experiment by Schwarz *et al.*^118^ The graph in Fig. 14(b) shows these extrema of gravity for this source mass arrangement. The freely falling test mass was then positioned in such a way that its trajectory covered the precise area of the saddle point. Lamporesi *et al.*^132^ used a similar arrangement. Furthermore, as Chen and Cook point out, a cylinder with a diameter equal to its length has almost the same gravitational field characteristics as the sphere.^43^ Another restriction is that perturbing forces should be avoided. Masses should therefore have a low magnetic and electric susceptibility and be made of electrically conductive materials in order to avoid electric charges.

## VI. SUMMARY AND OUTLOOK

**FIG. 18.**
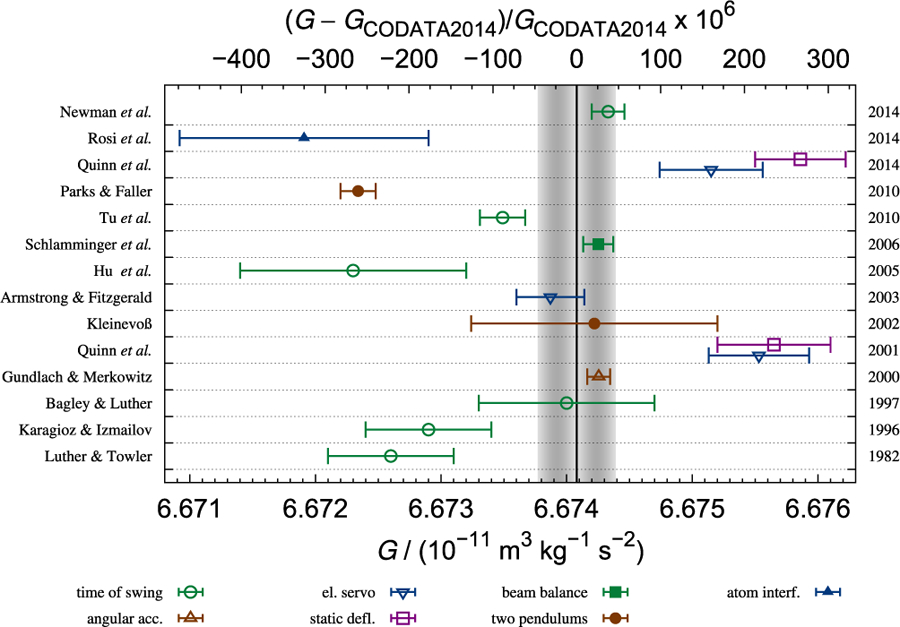
Recent measurements of *G* and their measurement uncertainties. The names on the left denote the principal authors and the numbers on the right denote the years when the results were published. The open symbols represent torsion balance experiments and the closed symbols represent measurements that were performed by other means. The vertical black line gives the CODATA-2014 recommended value, *G*_CODATA2014_, with its calculated uncertainty in gray.

Measuring *G* accurately is a challenging task. To determine *G*, a very carefully constructed setup is necessary since the gravitational force is about 38 orders of magnitude smaller than the electromagnetic force and since the gravitational interaction, unlike the electromagnetic interaction, cannot be shielded. A good indicator of the struggle to assign a value to *G* is given by the spread of the published values. Figure 18 shows the values and uncertainties of fourteen precision determinations of *G* published in the past 35 years. The smallest reported relative standard uncertainty is 14 × 10^−6^. However, the difference of the largest reported result to the smallest reported result exceeds 500 × 10^−6^, more than 30 times the smallest uncertainty.

In 2014, CODATA used a data set to determine the average value of *G* that was slightly different from the data set shown in Fig. 18. The two results from the different methods used by Quinn *et al.* were averaged to one data point for the 2001 and 2014 publications. In Fig. 18, the results were shown individually to emphasize the different modes of operation for torsion balances. Averaging both values results in significantly smaller relative uncertainties than either method due to the fact that the two determinations are anti-correlated, see Sec. IV A 2 b. The relative standard uncertainties of the averaged values are 41 × 10^−6^ and 25 × 10^−6^ for the values published in 2001 and 2014, respectively.

Faced with the large spread of the data compared to the typical uncertainties, the Task Group on Fundamental Constants decided to enlarge the reported uncertainty of all published results to include a common multiplicative factor before determining the uncertainty of the average value. One choice of multiplicative factor is the Birge ratio.^144,145^ The Birge ratio, *R*_B_, is named after Raymond Thayer Birge, an American physicist who published the first recommended set of fundamental constants in 1929.^146^ The Birge ratio is the square root of the sum of the squares of the normalized residuals divided by the number of degrees of freedom, i.e.,

(43) $$R_{\text{B}}=\sqrt{\frac{\chi^{2}}{N-1}}\;\;\text{with}\;\;\chi^{2}=\sum_{i=1}^{N}{\left(\frac{y_{i}-y_{\text{avg}}}{\sigma_{i}}\right)}^{2}.$$

The normalized residual of an experiment *i* is given by $\left(y_{i}-y_{\text{avg}}\right)/\sigma_{i}$, where *y_i_* and *σ_i_* are the published value and uncertainty of the experiment, respectively. Here, *y*_avg_ is the average value that can be obtained as a weighted average of the published data. The Birge ratio for the *G* data set is about five. This means that if every uncertainty is multiplied by a factor of five (replace *σ_i_* with 5*σ_i_* in the above equations), *χ*^2^ would be thirteen, the expectation value of a data set with fourteen measurements. The task group chose to use a slightly different expansion factor of 6.3 instead in order to reduce all normalized residuals below two.

Why is the scatter of the *G* data so large? Experimenters have devoted tremendous effort to investigating many possible contributions to the measurement uncertainty, but the complete data set reported is not statistically probable. In principle, there are three possibilities that can explain the observed inconsistency of the data:

1. Some or all of the experiments suffer from an unknown bias. A bias is a systematic effect that shifts the measured result from the true value by a predictable amount. It is normal for experiments to have biases. Usually, the experimenter determines the bias and applies a correction in such a way that the published value no longer has a bias, i.e., the experimenter’s best estimate of the true value. However, a bias may be present in the experiment that the experimenter is not aware of. In this case, the published result will differ from the true value by the size of the bias. For example, prior to 1995, the publication year of Kuroda’s article, the time-of-swing experiments suffered from a relative bias on the order of 1/*Q* because the experimenters were not aware that the inelastic properties of the spring affect the measurement result. After 1995, the experimenters tried to avoid the bias either by using a suspension with a large *Q* (e.g., Ref. 59) or by estimating the bias and applying a correction (e.g., Ref. 57).
2. Some or all of the experiments underestimate the relative uncertainty of the measurement. Hypothetically, all of the reported values of the measurements may be correct, but the uncertainties reported may be too small. If the true uncertainty were five times larger, the data set would be perfectly consistent (see the Birge ratio above). What could cause the experimenters to under-report the measurement uncertainty? We can say with certainty that this is not the intention of the experimenters, who usually spend a great deal of time and considerable resources in establishing a comprehensive uncertainty budget. In most *G* measurements, the time spent taking the actual measurement data is often much shorter than the time spent investigating the uncertainties.
  The principal problem is that the set of the known systematic and statistical effects is only a subset of all systematic and statistical effects that can perturb an experiment. Hence, regardless of how much effort is spent, the uncertainty budget can never be complete. There will always be an unknown uncertainty, often referred to as a dark uncertainty.^5^ The aim of the experimenter is to consider every conceivable effect that may change the result of the measurement in order to keep the dark uncertainty as small as possible (ideally, limiting it to a small fraction of the total reported uncertainty).
  Fortunately, however, as more experiments are conducted, more systematic effects are discovered. As our knowledge base of systematic effects grows, the sources of dark uncertainty will diminish and the scatter of future measurements will decrease. Eventually, the recommended value of *G* will converge to the true value.
3. The most exciting, yet least probable explanation is that new, unknown forms of physics can explain the variation in the data. This is a variant of the first point, with the difference being that the first point focuses on technical biases, while this point focuses on a bias that is more fundamental in nature. Over the years, several theories have been published that have attempted to explain the variation observed with the different experiments (e.g., Refs. 147 and 148). While attempts to formulate new theories are encouraged, the existing *G* data set is not the best data set to disprove such new theories. The experiments were all done with the intent of measuring *G*, and other variables may not have been strictly controlled. In most cases, it would be much better to conduct a dedicated experiment to disprove a certain theory. For example, if the hypothesis is that *G* depends on time, the best course of action is to build one experiment that is optimized to be precise and stable, but not necessarily accurate, because the true value of *G* would not be relevant for this test. The time series produced by such an experiment may be able to constrain a time-varying *G*, especially if all other variables are kept constant. A long data set of *G* measurements is available from the work of Karagioz.^51^ Theories of a time-varying *G* will necessarily predict a time variation of *g*, which contains *G*, unless there is a subtle canceling effect. Many institutes around the world measure *g* and unexplained variations of *g* within a year can be limited to well below one part in 10^9^.^149^

**FIG. 19.**
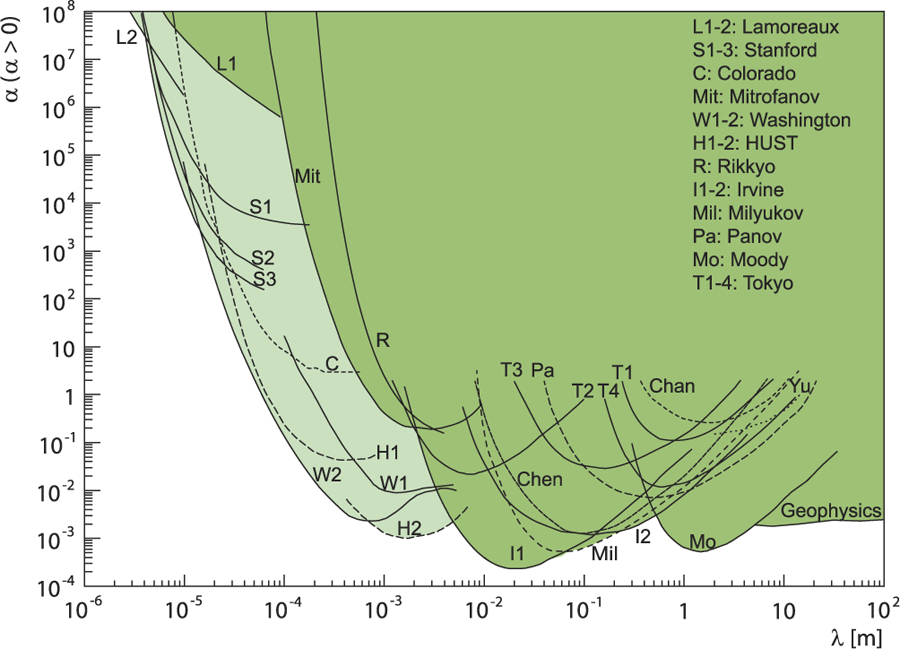
Current limits on the deviation of the gravitational law from an inverse square law. The deviations are parameterized as a Yukawa potential with an interaction range *λ* and a strength of *α* relative to Newtonian gravity, see text. Reprinted with permission from J. Murata, private communication (2017).^152^

An interesting possibility is a deviation of the inverse square law in the range of the laboratory experiments. A simple parameterization of a possible violation of the fifth force is the Yukawa potential. In the context here, this can be written as a distance-dependent constant of gravitation,

(44) $$G\left(r\right)=G_{\infty }\left(1+\alpha {\text{e}}^{-r/\lambda }\right),$$

where *α* denotes the strength of possible new interaction relative to gravity and *λ* is the typical range for the interaction. For r≫λ, *G* = *G*_∞_, and for r≪λ, *G* = *G*_∞_ (1 + *α*). For a review on the test of the inverse square law see Ref. 150. Figure 19 shows the current limits on *α* as a function of *λ*. Surprisingly, the exclusion is remarkably weak for the laboratory scale experiments discussed here with typical dimensions ranging from several centimeters to a few meters. Only *α* > 10^−3^ are ruled out. While a variation of *G* at ranges from a few centimeters to a few meters are not ruled out, they seem unlikely. Again, excluding a variation of *G* that has distances in the range of interest is best addressed in a dedicated experiment that compares the gravitational attraction on two length scales, rather than comparing the results of different *G* experiments.

Besides the technical and scientific issues discussed here and above, two additional facts hamper progress in measurements of *G*. First, most measurements are performed by small groups—publications with only two authors are not uncommon in this field. Very often, these groups measure *G* once and then move on to other experiments. The group led by Quinn is a notable exception. This makes it difficult to build *institutional memory*. In other words, every attempt to measure *G* starts from scratch and the investigators can only learn from the literature and not from their mentors. Second, experiments are not repeated. A core tenet of the scientific method is for results to be reproducible. Usually, a discovery is made and then verified by a second laboratory. However, no two identical *G* experiments have ever been repeated. For most researchers, it is more interesting and rewarding to invent a new method of measuring *G* than to repeat an existing measurement. However, repeating and independently assessing the uncertainty of the experiment is very important for this field.

In 2014, Quinn, Speake, and Luo invited many experimenters to discuss the situation of the *G* measurements.^58^ Later that year, NIST hosted another workshop to continue the discussion and proposed that a collaboration or consortium of several institutes—preferably national metrology institutes— be held in order to develop a common experiment.^152^ The idea was to make two identical setups so that two institutions could conduct the same experiment. Both were to give the same value for *G*, if not, the experimenters would have to search for the error until the results agreed within the single standard uncertainties. Although this would certainly not reveal all systematic errors, it would give more confidence in the result. Unfortunately, such a consortium has not yet been founded.

One positive outcome of both meetings was the formation of a “Big *G*” working group under the auspices of the International Union of Pure and Applied Physics (IUPAP).^153^ The purpose of this working group is to assist in resolving the discrepancy present in *G* measurements. An additional function of the working group could be to provide institutional memory, mentoring, and advice for new experiments.

In addition, the International Committee for Weights and Measures (CIPM) decided in its November 2014 meeting to establish a consortium of national metrology institutes to facilitate new work aimed at resolving the present disagreement among measurements of the Newtonian constant of gravitation.^154^

Given the current situation in the measurement of *G*, it is difficult to see how our knowledge of *G* can be improved, for example, *χ*^2^ will not decrease by adding new experiments, as it is a sum of squares and can increase only with new data. The Birge ratio can decrease by increasing $\sqrt{N-1}$ in the denominator; however, this will be a slow process. If an additional 13 experiments are performed (which could take another 30 years if past experiments are an indication), *R*_B_ can be reduced by a factor 1.4 if the values are close to the current average value. It is equally difficult to see how the multiplicative factor that CODATA used to bring all normalized residuals below two can be decreased. Thus, decreasing the current uncertainty assigned to the recommended value of *G* does not seem to be possible—at least, not in the foreseeable future.

However, re-evaluating or repeating experiments that have already been performed may provide insights into hidden biases or dark uncertainty. NIST has the unique opportunity to repeat the experiment of Quinn *et al.*^72^ with an almost identical setup. By mid-2018, NIST researchers will publish their results and assign a number as well as an uncertainty to their value. The same researchers have also acquired the equipment of Parks and Faller^103^ although there are no immediate plans to repeat this experiment. Securing the equipment to prevent it from being lost is an important first step. Because these two experiments span almost the whole range of all *G* values, having both of them is an important asset. The relevance of repeating previous experiments after so many years may be called into question, as technology has changed, and an improved setup should be possible. One might liken this situation to ascending Mount Everest using the equipment of Sir Edmund Hillary. However, the lessons learned by realizing a situation from another point of view (i.e., following in the footsteps of others) can prove to be highly instructive. The experience that experimenters have acquired to date using new equipment may help to identify unconsidered systematical errors.^151^

## VII. CONCLUSIONS

The measurement of the constant of gravitation, “Big *G*,” is still one of the most challenging of all experiments. Being the second fundamental constant ever measured, it remains the fundamental physical constant with the highest measurement uncertainty. We have given an overview of recent and historic measurements in order to show the different experimental approaches that have been taken over the past 200 years. Furthermore, ongoing efforts are in progress to unveil the origins of large discrepancies in recent measurements. The National Metrology Institute of the United States, NIST, is repeating one “Big *G*” measurement conducted by another group.

It is hoped that the quest for a more accurate “Big *G*” will not cease, as science has yet to devise a solution to the mystery of why “Big *G*” measurements do not converge.

## Figures and Tables

**TABLE I.** Parameters of torsion balances used in recent determinations of *G*. Unless otherwise noted, the torsion fibers have a circular cross section.

Measurement	*κ*(N m rad^−1^)	*f _o_* (mHz)	*Q*	Remarks	References
Luther and Towler	3.9 × 10^−10^	2.8	2 × 10^4^	Quartz torsion fiber	49 and 50
Karagioz and Izmailov	3.1 × 10^−10^	0.5	2 × 10^4^	Tungsten fiber	51
Bagley and Luther	1.2 × 10^−9^	4.9	9.5 × 10^2^	Tungsten fiber	52
	1.2 × 10^−9^	4.9	4.9 × 10^2^	Gold-coated tungsten fiber	
Gundlach and Merkowitz	3.5 × 10^−9^	4.0	4 × 10^3^	Tungsten fiber, values not given	53 and 54
Quinn *et al.*	2.1 × 10^−4^	8.0	3 × 10^5^	Torsion strip made from Cu-1.8% Be	55
Armstrong and Fitzgerald	n/a	n/a	n/a	Rectangular tungsten fiber	56
Tu *et al.*	6.3 × 10^−9^	1.9	1.7 × 10^3^	Tungsten fiber	57
Quinn	2.1 × 10^−4^	8.0	3 × 10^5^	Same as 2001 experiment	58
Newman *et al.*	3.1 × 10^−9^	7.4	8.2 × 10^4^	Fiber 1: CuBe	59
	3.4 × 10^−9^	7.7	1.2 × 10^5^	Fiber 2: CuBe annealed	
	3.1 × 10^−9^	8.8	1.8 × 10^5^	Fiber 3: Al5056	

**TABLE II.** Parameters of time-of-swing measurements that were published in recent years. The second column gives the period of the torsion pendulum with the field masses in the “far” position. The numbers in the third column show the amount by which the period shortens when the field masses are near. These numbers provide an idea of the required time resolution. The ratio $\Delta \omega^{2}/\omega^{2}$ is identical to the ratio of the gravitational spring constant to that of the torsion fiber. Karagioz and Izmailov used several different torsion balances during the course of their experiment, which began in 1982 and is still ongoing today. The entry shown is from what Karagioz and Izmailov refer to as “Version 1” in Ref. 51. The change in period depends on the amplitude of the torsion pendulum. The experiment carried out by Newman *et al.* used different amplitudes and fibers. The change in period ranges from 0.2 ms to 1.7 ms.

Measurement	*T* (s)	∆*T*	$\frac{\omega_{\text{n}}^{2}-\omega_{\text{f}}^{2}}{\omega_{\text{f}}^{2}}$
Luther and Towler	328	2.8 s	3.4 × 10^−2^
Karagioz and Izmailov	2077	n/a	n/a
Bagley and Luther	205	2.5 s	2.4 × 10^−2^
Tu *et al.*	536	3.3 s	1.2 × 10^−2^
Newman *et al.*	134	1.7 ms	2.5 × 10^−5^

**TABLE III.** Field masses used in determinations of *G*. Adapted from Ref. 133.

Field mass (total) (kg)	Material	Geometry	Measurement principle	References
1.6	Stainless steel	Spheres	Torsion balance	57
21	Tungsten	Spheres	Torsion balance	50
33	Stainless steel	Spheres	Torsion balance	53
45	Cu 0.7% Te	Cylinders	Torsion balance	72
118	Copper	Rings	Torsion balance	59
480	Tungsten	Cylinders	Double pendulum	103
516	Tungsten	Cylinders	Atom gravimeter	132
521	Tungsten alloy	Cylinders	Free-fall gravimeter	118
1 152	Brass	Cylinders	Double pendulum	102
5 775	Lead	Sphere	Beam balance	106
13 520	Mercury	Cylinder tank	Beam balance	114
100 000	Lead	Rectangular block	Beam balance	109

## References

[1] Newton I, Philosophiae Naturalis Principia Mathematica (Sumptibus Societatis, 1687).

[2] Einstein A, Die Feldgleichungen der Gravitation, Sitzungsberichte der Königlich Preußischen Akademie der Wissenschaften (Berlin, 1915), pp. 844–847.

[3] Will CM, “The confrontation between general relativity and experiment,” Living Rev. Relativ 9(1), 3 (2006). 28179873 10.12942/lrr-2006-3 PMC5256066

[4] Gillies GT, “The Newtonian gravitational constant: An index of measurements,” Metrologia 24(S), 1 (1987).

[5] Thompson M and Ellison SLR, “Dark uncertainty,” Accredit. Qual. Assur 16(10), 483 (2011).

[6] Branscomb LM, “Views: Integrity in science: Much of the problem of honor–or lack of honor–in science stems not from malice but from self-deception,” Am. Sci 73(5), 421–423 (1985).

[7] Cohen ER and DuMond JWM, “Our knowledge of the fundamental constants of physics and chemistry in 1965,” Rev. Mod. Phys 37, 537 (1965).

[8] Gillies GT, “The Newtonian gravitational constant: Recent measurements and related studies,” Rep. Prog. Phys 60(2), 151 (1997).

[9] Sagitov MU, “Current status of determinations of the gravitational constant and the mass of the Earth,” Sov. Astron. - AJ 13, 712–718 (1970).

[10] De Boer H, “Experiments relating to the Newtonian gravitational constant,” in: Precision Measurement and Fundamental Constants, edited by Taylor BN and Phillips WD (National Bureau of Standards Publication 617, U.S. Government Printing Office, Washington, D.C., 1984), pp. 561–572.

[11] Poynting JH, Gravitation, Volume XII–Gichtel to Harmonium of the Encyclopaedia Britannica (Cambridge University Press, 1910), pp. 384–389.

[12] Mackenzie AS, Newton I, Bouguer MP, and Cavendish H, The Laws of Gravitation: Memoirs by Newton, Bouguer and Cavendish, Together With Abstracts of Other Important Memoirs (American Book Company, 1900).

[13] Cook A, “Experiments on gravitation,” Rep. Prog. Phys 51(5), 707 (1988).

[14] Hu Z-K, Liu Q, and Luo J, “Determination of the gravitational constant G,” Front. Phys. China 1(4), 449–457 (2006).

[15] Burgess GK, “Recherches sur la constante de gravitation,” Ph.D. thesis, Librairie Scientifique A. Hermann, L’Université de Paris, 1901.

[16] Luo J and Hu Z-K, “Status of measurement of the Newtonian gravitational constant G,” Classical Quantum Gravity 17(12), 2351 (2000).

[17] Soffel HC, Phillipp Johann von Jolly (1809–1884) In DGG Mitteilungen, DGG, 2009.

[18] Bell RE and Hansen RO, “The rise and fall of early oil field technology: The torsion balance gradiometer,” in The Leading Edge (Society of Exploration Geophysicists, 1998), pp. 81–83.

[19] Mohr PJ, Newell DB, and Taylor BN, “CODATA recommended values of the fundamental constants: 2014,” Rev. Mod. Phys 88, 035009 (2016). 10.1103/RevModPhys.93.025010 PMC9890581 36733295

[20] Evenson KM, Wells JS, Petersen FR, Danielson BL, Day GW, Barger RL, and Hall JL, “Speed of light from direct frequency and wavelength measurements of the methane-stabilized laser,” Phys. Rev. Lett 29, 1346–1349 (1972).

[21] Mohr PJ and Taylor BN, “CODATA recommended values of the fundamental physical constants: 1998,” Rev. Mod. Phys 72, 351 (2000). 10.1103/RevModPhys.93.025010 PMC9890581 36733295

[22] Mohr PJ and Taylor BN, “CODATA recommended values of the fundamental physical constants: 2002,” Rev. Mod. Phys 77(1), 1–107 (2005). 10.1103/RevModPhys.93.025010 PMC9890581 36733295

[23] Mohr PJ, Taylor BN, and Newell DB, “CODATA recommended values of the fundamental physical constants: 2006,” Rev. Mod. Phys 80, 633 (2008). 10.1103/RevModPhys.93.025010 PMC9890581 36733295

[24] Mohr PJ, Taylor BN, and Newell DB, “CODATA recommended values of the fundamental physical constants: 2010,” Rev. Mod. Phys 84, 1527 (2012). 10.1103/RevModPhys.93.025010 PMC9890581 36733295

[25] Cohen ER and Taylor BN, “The 1986 adjustment of the fundamental physical constants,” Rev. Mod. Phys 59, 1121 (1987). 10.1103/RevModPhys.93.025010 PMC9890581 36733295

[26] Cohen ER and Taylor BN, “The 1973 least-squares adjustment of the fundamental constants,” J. Phys. Chem. Ref. Data 2, 663 (1973).

[27] Cavendish H, “Experiments to determine the density of the Earth. By Henry Cavendish, Esq. F. R. S. and A. S.,” Philos. Trans. R. Soc. London 88, 469–526 (1798).

[28] Tomilin KA, “Natural systems of units. To the centenary anniversary of the Planck system,” in Proceedings of the XXII Workshop on High Energy Physics and Field Theory (CiteSeer, 1999).

[29] König A and Richarz F, “Eine neue Methode zur Bestimmung der Gravitationsconstante,” Ann. Phys 260(4), 664–668 (1885).

[30] Planck M, “Ueber irreversible Strahlungsvorgänge,” Ann. Phys 306(1), 69–122 (1900).

[31] Kolosnitsyn NI, “Calibration of gravitational gradiometers using gradient gauges,” Meas. Tech 35(12), 1443–1447 (1992).

[32] McQueen HWS, “Independence of the gravitational constant from gross Earth data,” Phys. Earth Planet. Inter 26(3), P6–P9 (1981).

[33] Sahu KC, Anderson J, Casertano S, Bond HE, Bergeron P, Nelan EP, Pueyo L, Brown TM, Bellini A, Levay ZG, Sokol J, Dominik M, Calamida A, Kains N, and Livio M, “Relativistic deflection of background starlight measures the mass of a nearby white dwarf star,” Science 356(6342), 1046–1050 (2017). 28592430 10.1126/science.aal2879

[34] Wikipedia. Orders of Magnitude (Mass)—Wikipedia, The Free Encyclopedia, 2017. Online; accessed 3 November 2017.

[35] Brandes HW, Gmelin L, Horner JC, Muncke GW, and Pfaff CH, Johann Samuel Traugott Gehler’s Physikalisches Wörterbuch–Zweiter Band C–D (Schwickert, Leipzig, 1826).

[36] Kuroda K, “Does the time-of-swing method give a correct value of the Newtonian gravitational constant?,” Phys. Rev. Lett 75(15), 2796–2798 (1995). 10059407 10.1103/PhysRevLett.75.2796

[37] Armano M , “Sub-femto-g free fall for space-based gravitational wave observatories: LISA pathfinder results,” Phys. Rev. Lett 116(23), 231101 (2016). 27341221 10.1103/PhysRevLett.116.231101

[38] Sanders AJ and Gillies GT, “A comparative survey of proposals for space-based determination of the gravitational constant G,” Riv. Nuovo Cimentodel 19(2), 1–54 (1996).

[39] Melnikov VN, “Centenary of Einstein’s general relativity. Its present extensions,” Gravitation Cosmol 22(2), 80–96 (2016).

[40] See https://www.zarm.uni-bremen.de/drop-tower.html for “ZARM–Center of Applied Space Technology and Microgravity”; accessed 19 August 2016.

[41] Reasenberg RD and Phillips JD, “A weak equivalence principle test on a suborbital rocket,” Classical Quantum Gravity 27(9), 095005 (2010).

[42] Haddad D, Seifert F, Chao LS, Li S, Newell DB, Pratt JR, Williams C, and Schlamminger S, “Invited article: A precise instrument to determine the Planck constant, and the future kilogram,” Rev. Sci. Instrum 87(6), 061301 (2016). 27370418 10.1063/1.4953825 PMC7063581

[43] Chen YT and Cook A, Gravitational Experiments in the Laboratory (Cambridge University Press, 2005).

[44] Gillies GT and Ritter RC, “Torsion balances, torsion pendulums, and related devices,” Rev. Sci. Instrum 64, 283 (1993).

[45] Jones RV and Richards JCS, “Recording optical lever,” J. Sci. Instrum 36(2), 90 (1959).

[46] Cowsik R, Srinivasan R, Kasturirengan S, Senthil Kumar A, and Wagoner K, “Design and performance of a sub-nanoradian resolution autocollimating optical lever,” Rev. Sci. Instrum 78(3), 035105 (2007). 17411215 10.1063/1.2714044

[47] Arp TB, Hagedorn CA, Schlamminger S, and Gundlach JH, “A reference-beam autocollimator with nanoradian sensitivity from mHz to kHz and dynamic range of 107,” Rev. Sci. Instrum 84(9), 095007 (2013). 24089858 10.1063/1.4821653

[48] Saulson PR, “Thermal noise in mechanical experiments,” Phys. Rev. D 42, 2437–2445 (1990). 10.1103/physrevd.42.2437 10013112

[49] Luther GG and Towler WR, “Precision measurement and fundamental constants II,” in: Atomic Masses and Fundamental Constants, edited by Sanders JH and Wapstra AH (Plenum, New York, 1981), Vol. 5, pp. 545–551.

[50] Luther GG and Towler RW, “Redetermination of the Newtonian gravitational constant G,” Phys. Rev. Lett 48(3), 121 (1982).

[51] Karagioz OV and Izmailov VP, “Measurement of the gravitational constant with a torsion balance,” Meas. Tech 39(10), 979 (1996).

[52] Bagley CH and Luther GG, “Preliminary results of a determination of the Newtonian constant of gravitation: A test of the Kuroda hypothesis,” Phys. Rev. Lett 78, 3047–3050 (1997).

[53] Gundlach JH and Merkowitz SM, “Measurement of Newton’s constant using a torsion balance with angular acceleration feedback,” Phys. Rev. Lett 85, 2869–2872 (2000). 11005956 10.1103/PhysRevLett.85.2869

[54] Gundlach J, private communication (2017).

[55] Quinn TJ, Speake C, Richman SJ, Davis RS, and Picard A, “A new determination of G using two methods,” Phys. Rev. Lett 87, 111101 (2001). 11531510 10.1103/PhysRevLett.87.111101

[56] Armstrong TR and Fitzgerald MP, “New measurements of G using the measurement standards laboratory torsion balance,” Phys. Rev. Lett 91, 201101 (2003). 14683348 10.1103/PhysRevLett.91.201101

[57] Tu L-C, Li Q, Wang Q-L, Shao C-G, Yang S-Q, Liu L-X, Liu Q, and Luo J, “New determination of the gravitational constant G with time-of-swing method,” Phys. Rev. D 82(2), 022001 (2010). 10.1103/PhysRevLett.102.240801 19658992

[58] Quinn T, “Outcome of the Royal Society meeting on G held at Chicheley Hall on 27 and 28 February 2014 to discuss ‘The Newtonian constant of gravitation, a constant too difficult to measure?’,” Philos. Trans. R. Soc., A 372(2026), 20140286 (2014). 10.1098/rsta.2014.0286 25202005

[59] Newman R, Bantel M, Berg E, and Cross W, “A measurement of G with cryogenic torsion pendulum,” Philos. Trans. R. Soc., A 372, 20140025 (2014). 10.1098/rsta.2014.0025 25202000

[60] Heptonstall A, Barton MA, Bell AS, Bohn A, Cagnoli G, Cumming A Grant A, Gustafson E, Hammond GD, Hough J, Jones R, Kumar R, Lee K, Martin IW, Robertson NA, Rowan S, Strain KA, and Tokmakov KV, “Enhanced characteristics of fused silica fibers using laser polishing,” Classical Quantum Gravity 31(10), 105006 (2014).

[61] Cumming AV, Cunningham L, Hammond GD, Haughian K, Hough J, Kroker S, Martin IW, Nawrodt R, Rowan S, Schwarz C, and van Veggel AA, “Silicon mirror suspensions for gravitational wave detectors,” Classical Quantum Gravity 31(2), 025017 (2014).

[62] Ugolini D, Girard M, Harry GM, and Mitrofanov VP, “Discharging fused silica test masses with ultraviolet light,” Phys. Lett. A 372(36), 5741–5744 (2008).

[63] Pollack SE, Turner MD, Schlamminger S, Hagedorn CA, and Gundlach JH, “Charge management for gravitational-wave observatories using UV LEDs, Phys. Rev. D 81, 021101 (2010).

[64] Hagedorn CA, Schlamminger S, and Gundlach JH, “Quality factors of bare and metal-coated quartz and fused silica torsion fibers,” AIP Conf. Proc 873, 189–193 (2006).

[65] Bantel MK and Newman RD, “High precision measurement of torsion fiber internal friction at cryogenic temperatures,” J. Alloys Compd 310(1–2), 233–242 (2000).

[66] Feder HJS and Feder J, “Self-organized criticality in a stick-slip process,” Phys. Rev. Lett 66, 2669–2672 (1991). 10043581 10.1103/PhysRevLett.66.2669

[67] Richman SJ, Quinn TJ, Speake CC, and Davis RS, “Preliminary determination of G using the BIPM torsion strip balance,” Meas. Sci. Technol 10, 460 (1999).

[68] Boys CV, “On the Cavendish experiment,” Proc. R. Soc. London 46, 253–268 (1889).

[69] Sutton CM and Clarkson MT, “A general approach to comparisons in the presence of drift,” Metrologia 30(5), 487 (1994).

[70] Gläser M, “Cycles of comparison measurements, uncertainties and efficiencies,” Meas. Sci. Technol 11(1), 20 (2000).

[71] Swanson EH and Schlamminger S, “Removal of zero-point drift from AB data and the statistical cost,” Meas. Sci. Technol 21(11), 115104 (2010).

[72] Quinn T, Parks H, Speake C, and Davis R, “Improved determination of G using two methods,” Phys. Rev. Lett 111, 101102 (2013). 25166649 10.1103/PhysRevLett.111.101102

[73] Luo J, Hu Z-K, Fu X-H, Fan S-H, and Tang M-X, “Determination of the Newtonian gravitational constant G with a nonlinear fitting method,” Phys. Rev. D 59, 042001 (1998).

[74] Hu Z-K, Guo J-Q, and Luo J, “Correction of source mass effects in the HUST-99 measurement of G,” Phys. Rev. D 71, 127507 (2005).

[75] Reich F, “Neue Versuche mit der Dehwaage,” Abh. Math.-Phys. Cl. Königliche Sächsischen Ges. Wiss 1, 384 (1852).

[76] Brandes HW, Gmelin L, Horner JC, Muncke GW, and Pfaff CH, Johann Samuel Traugott Gehler’s Physikalisches Wörterbuch–Dritter B and E (Schwickert, Leipzig, 1827).

[77] Brandes HW, “Theoretische Untersuchungen über die Oscillationen der Drehwaage bei Cavendish’s Versuchen über die Attraction kleiner Massen,” Mag. Neuesten Zustand Naturkd 12, 300–310 (1806).

[78] Braun C, Die Gravitations-Constante, die Masse und Mittlere Dichte der Erde Nach Einer Neuen Experimentellen Bestimmung Von Carl Braun: (Mit 3 Tafeln u. 8 Textfiguren) (Separatabdruck Aus d. 64. Bande d. Denkschriften d. Math.-Naturwissensch. Classe d. K. Akad. d. Wissenschaften) (Carl Gerolds Sohn, 1896).

[79] Newman RD and Bantel MK, “On determining G using a cryogenic torsion pendulum,” Meas. Sci. Technol 10, 445 (1999).

[80] Roll PG, Krotkov R, and Dicke RH, “The equivalence of inertial and passive gravitational mass,” Ann. Phys 26, 442 (1964).

[81] de Boer H, Haars H, Michaelis W, and Schlimme E, “Quadrantenelektrometer als Drehmomentmesser für kleine Drehmomente,” Feinwerktech. Messtech 88, 237–241 (1980).

[82] de Boer H, Haars H, and Michaelis W, “A new experiment for the determination of the Newtonian gravitational constant,” Metrologia 24(4), 171 (1987).

[83] Fitzgerald MP and Armstrong TR, “Newton’s gravitational constant with uncertainty less than 100 ppm,” IEEE Trans. Instrum. Meas 44, 494 (1995).

[84] Fitzgerald MP, Armstrong RB, Hurst TR, and Corney AC, “A method to measure Newton’s gravitational constant,” Metrologia 31, 301 (1994).

[85] Armstrong TR and Fitzgerald MP, “An autocollimator based on the laser head of a compact disc player,” Meas. Sci. Technol 3, 1072 (1992).

[86] Quinn T, Speake CC, Parks H, and Davis R, “The BIPM measurements of the Newtonian constant of gravitation, G,” Philos. Trans. R. Soc., A 372, 20140032 (2014). 10.1098/rsta.2014.0032 25201995

[87] Jones RV and Richards JCS, “The design and some applications of sensitive capacitance micrometers,” J. Phys. E: Sci. Instrum 6, 589 (1973).

[88] Jackson JD, Classical Electrodynamics, 3rd ed. (Wiley, New York, NY, 1999).

[89] Speake CC, “Newton’s constant and the twenty-first century laboratory,” Philos. Trans. R. Soc., A 363, 2265 (2005). 10.1098/rsta.2005.1643 16147509

[90] Michaelis W, Haars H, and Augustin R, “A new precise determination of Newton’s gravitational constant,” Metrologia 32(4), 267 (1995).

[91] Michaelis W, Melcher J, and Haars H, “Supplementary investigations to PTB’s evaluation of G,” Metrologia 41, L29 (2004).

[92] Shaw GA, Stirling J, Kramar JA, Moses A, Abbott P, Steiner R, Koffman A, Pratt JR, and Kubarych ZJ, “Milligram mass metrology using an electrostatic force balance,” Metrologia 53, A86 (2016).

[93] Rose RD, Parker HM, Lowry RA, Kuhlthau AR, and Beams JW, “Determination of the gravitational constant G,” Phys. Rev. Lett 23(12), 655 (1969).

[94] Su Y, Heckel BR, Adelberger EG, Gundlach JH, Harris M, Smith GL, and Swanson HE, “New tests of the universality of free fall,” Phys. Rev. D 50, 3614 (1994). 10.1103/physrevd.50.3614 10018005

[95] Adelberger EG, Collins NA, and Hoyle CD, “Analytic expressions for gravitational inner multipole moments of elementary solids and for the force between two rectangular solids,” Classical Quantum Gravity 23, 125 (2006).

[96] Adelberger EG, Collins NA, and Hoyle CD, “Analytic expressions for gravitational inner multipole moments of elementary solids and for the force between two rectangular solids–erratum,” Classical Quantum Gravity 23, 5463 (2006).

[97] Meyer TH, Roman DR, and Zilkoski DB, “What does height really mean? Part I: Introduction,” Surv. Land Inf. Sci 64(4), 223–233 (2004), available at http://opencommons.uconn.edu/thmeyerarticles/2.

[98] Bouguer P, La Figure de la Terre (1749).

[99] Maskelyne N, “An account of observations made on the mountain Schehallien for finding its attraction. By the Rev. Nevil Maskelyne, B. D. F. R. S. and Astronomer Royal,” Philos. Trans. R. Soc. London 65, 500–542 (1775).

[100] De Marchi A, “A frequency metrology approach to Newtonian constant G determination using a pair of extremely high Q simple pendulums in free decay,” J. Phys.: Conf. Ser 723, 012046 (2016).

[101] Kleinevoß U, Meyer H, Schumacher A, and Hartmann S, “Absolute measurement of the Newtonian force and a determination of G,” Meas. Sci. Technol 10(6), 492 (1999).

[102] Kleinevoß U, “Bestimmung der Newtonschen Gravitationskonstanten G,” WUB-DISS 2002–2, Ph.D. thesis, University of Wuppertal, Germany, 2002.

[103] Parks HV and Faller JE, “Simple pendulum determination of the gravitational constant,” Phys. Rev. Lett 105(11), 110801 (2010). 20867560 10.1103/PhysRevLett.105.110801

[104] Parks HV and Faller JE, “A simple pendulum laser interferometer for determining the gravitational constant,” Philos. Trans. R. Soc., A 372(2026), 20140024 (2014). 10.1098/rsta.2014.0024 PMC4173269 25201994

[105] von Jolly P, “Die anwendung der waage auf probleme der gravitation: Erste abhandlung,” Ann. Phys 241, 112–134 (1878).

[106] von Jolly P, “Die Anwendung der Waage auf Probleme der gravitation: Zweite Abhandlung,” Ann. Phys 250, 331–355 (1881).

[107] von Voit C, “Philipp Johann Gustav von Jolly,” Sitzungsber. Math.-Phys. Cl. Akad. Wiss. Münch 15, 119–136 (1885).

[108] Graetz L, Die Physik (Max Planck Institute for the History of Science, Leipzig, 1917), S. 66.

[109] Richarz F and Krigar-Menzel O, “Gravitationsconstante und mittlere dichtigkeit der erde, bestimmt durch wägungen,” Ann. Phys 302(10), 177–193 (1898).

[110] Poynting JH, “On a method of using the balance with great delicacy, and on its employment to determine the mean density of the Earth,” Proc. R. Soc. London 28(190–195), 1–35 (1878).

[111] Speake CC, “A beam balance method for determining the Newtonian gravitational constant,” Ph.D. thesis, University of Cambridge, England, 1983.

[112] Speake CC and Gillies GT, “The beam balance as a detector in experimental gravitation,” Proc. R. Soc. A 414, 315 (1987).

[113] Schlamminger S, Holzschuh E, and Kündig W, “Determination of the gravitational constant with a beam balance,” Phys. Rev. Lett 89, 161102 (2002). 12398712 10.1103/PhysRevLett.89.161102

[114] Schlamminger S, Holzschuh E, Kündig W, Nolting F, Pixley RE, Schurr J, and Straumann U, “Measurement of Newton’s gravitational constant,” Phys. Rev. D 74(8), 082001 (2006).

[115] Cornaz A, Hubler B, and Kündig W, “Determination of the gravitational constant at an effective interaction distance of 112 m,” Phys. Rev. Lett 72(8), 1152 (1994). 10056636 10.1103/PhysRevLett.72.1152

[116] Hubler B, Cornaz A, and Kündig W, “Determination of the gravitational constant with a lake experiment: New constraints for non-Newtonian gravity,” Phys. Rev. D 51(8), 4005 (1995). 10.1103/physrevd.51.4005 10018875

[117] Schlamminger S, Pixley RE, Nolting F, Schurr J, and Straumann U, “Reflections on a measurement of the gravitational constant using a beam balance and 13 tons of mercury,” Philos. Trans. R. Soc., A 372(2026), 20140027 (2014). 10.1098/rsta.2014.0027 25202003

[118] Schwarz JP, Robertson DS, Niebauer TM, and Faller JE, “A free-fall determination of the Newtonian constant of gravity,” Science 282, 2230–2234 (1998). 9856940 10.1126/science.282.5397.2230

[119] Timmen L, “Precise definition of the effective measurement height of free-fall absolute gravimeters,” Metrologia 40(2), 62 (2003).

[120] Rothleitner C and Svitlov S, “On the evaluation of systematic effects in atom and corner-cube absolute gravimeters,” Phys. Lett. A 376(12), 1090–1095 (2012).

[121] Niebauer T, “3.03—Gravimetric methods—Absolute and relative gravity meter: Instruments concepts and implementation, in Treatise on Geophysics, 2nd ed., edited by Schubert G (Elsevier, Oxford, 2015), pp. 37–57.

[122] Kasevich M and Chu S, “Atomic interferometry using stimulated Raman transitions,” Phys. Rev. Lett 67(2), 181–184 (1991). 10044515 10.1103/PhysRevLett.67.181

[123] Kasevich M and Chu S, “Measurement of the gravitational acceleration of an atom with a light-pulse atom interferometer,” Appl. Phys. B: Photophys. Laser Chem 54, 321–332 (1992).

[124] Cronin AD, Schmiedmayer J, and Pritchard DE, “Optics and interferometry with atoms and molecules,” Rev. Mod. Phys 81, 1051–1129 (2009).

[125] Rosi G, “Challenging the big G measurement with atoms and light,” J. Phys. B: At., Mol. Opt. Phys 49(20), 202002 (2016).

[126] Fixler JB, Foster GT, McGuirk JM, and Kasevich MA, “Atom interferometer measurement of the Newtonian constant of gravity,” Science 315(5808), 74–77 (2007). 17204644 10.1126/science.1135459

[127] Rosi G, Sorrentino F, Cacciapuoti L, Prevedelli M, and Tino GM, “Precision measurement of the Newtonian gravitational constant using cold atoms,” Nature 510(7506), 518–521 (2014). 24965653 10.1038/nature13433

[128] Rothleitner C and Francis O, “Measuring the Newtonian constant of gravitation with a differential free-fall gradiometer: A feasibility study,” Rev. Sci. Instrum 85(4), 044501 (2014). 24784631 10.1063/1.4869875

[129] Rothleitner C and Francis O, “On the influence of the rotation of a corner cube reflector in absolute gravimetry,” Metrologia 47(5), 567 (2010).

[130] Rothleitner C, Svitlov S, Mérimechè H, and Wang LJ, “A method for adjusting the centre of mass of a freely falling body in absolute gravimetry,” Metrologia 44(3), 234 (2007).

[131] Rosi G, Cacciapuoti L, Sorrentino F, Menchetti M, Prevedelli M, and Tino GM, “Measurement of the gravity-field curvature by atom interferometry,” Phys. Rev. Lett 114, 013001 (2015). 25615464 10.1103/PhysRevLett.114.013001

[132] Lamporesi G, Bertoldi A, Cacciapuoti L, Prevedelli M, and Tino GM, “Determination of the Newtonian gravitational constant using atom interferometry,” Phys. Rev. Lett 100(5), 050801 (2008). 18352354 10.1103/PhysRevLett.100.050801

[133] Gillies GT and Unnikrishnan CS, “The attracting masses in measurements of G: An overview of physical characteristics and performance,” Philos. Trans. R. Soc., A 372(2026), 20140022 (2014). 10.1098/rsta.2014.0022 25201999

[134] Arnet F, Klingelé E, and Straub C, Bestimmung der Gravitationskonstanten G in der Staumauer Gigerwald (IGP, 1995).

[135] Zumberge MA, Hildebrand JA, Stevenson JM, Parker RL, Chave AD, Ander ME, and Spiess FN, “Submarine measurement of the Newtonian gravitational constant,” Phys. Rev. Lett 67(22), 3051 (1991). 10044628 10.1103/PhysRevLett.67.3051

[136] Nicolaus RA and Bartl G, “Spherical interferometry for the characterization of precision spheres,” Surf. Topogr.: Metrol. Prop 4(3), 034007 (2016).

[137] Fujii K, Bettin H, Becker P, Massa E, Rienitz O, Pramann A, Nicolaus A, Kuramoto N, Busch I, and Borys M, “Realization of the kilogram by the XRCD method,” Metrologia 53(5), A19 (2016).

[138] Beams JW, Phys. Today 24(5), 34 (1971).

[139] DIN 5401 Rolling bearings—Balls for rolling bearings and general industrial use, 2002–2008.

[140] Lamporesi G, Bertoldi A, Cecchetti A, Duhlach B, Fattori M, Malengo A, Pettorruso S, Prevedelli M, and Tino GM, “Source mass and positioning system for an accurate measurement of G,” Rev. Sci. Instrum 78(7), 075109 (2007). 17672795 10.1063/1.2751090

[141] Meeß R, private communication (2017).

[142] Faller JE and Koldewyn WA, “A prototype measurement of the Newtonian gravitational constant using an active magnetic suspension torsion fiber,” in Precision Measurement and Gravity Experiment (National Tsing Hua University, 1983), p. 541.

[143] Chen YT and Cook A, “Mathematical behaviour around the stationary point of a gravitational ring,” Phys. Lett. A 138(8), 378–380 (1989).

[144] Birge RT, “The calculation of errors by the method of least squares,” Phys. Rev 40, 207 (1932).

[145] Bodnar O and Elster C, “On the adjustment of inconsistent data using the Birge ratio,” Metrologia 51(5), 516 (2014).

[146] Birge RT, “Probable values of the general physical constants,” Mod. Rev. Phys 1, 1 (1929).

[147] Anderson JD, Schubert G, Trimble V, and Feldman MR, “Measurements of Newton’s gravitational constant and the length of day,” Europhys. Lett 110, 10002 (2015).

[148] Mbelek JP and Lachièze-Rey M, “Possible evidence from laboratory measurements for a latitude and longitude dependence,” Gravitation Cosmol 8, 331 (2002).

[149] Crossley D, Hinderer J, and Riccardi U, “The measurement of surface gravity,” Rep. Prog. Phys 76(4), 046101 (2013). 23503405 10.1088/0034-4885/76/4/046101

[150] Murata J and Tanaka S, “A review of short-range gravity experiments in the LHC era,” Classical Quantum Gravity 32, 033001 (2015).

[151] Murata J, private communication (2017).

[152] See https://www.nist.gov/programs-projects/newtonian-constant-gravitation-international-consortium for “Newtonian constant of gravitation international consortium”; accessed 29 May 2017.

[153] See http://iupap.org/working-groups/wg13-newtonian-constant-of-gravitation/ for “WG13: Newtonian constant of gravitation.”

[154] See https://www.bipm.org/utils/en/pdf/CIPM/CIPM2014-II-Decisions-EN.pdf for “Decision CIPM/103–43”; accessed 29 May 2017.

